# Haplotype-resolved regulation of secondary metabolism in cannabis

**DOI:** 10.1093/hr/uhag159

**Published:** 2026-04-28

**Authors:** Sen Cai, Zhiyuan Zhang, Xiang Li, Yucheng Zhao, Chengwei Lin, Manru Huang, Mingwei Shen, Deyong Ao, Yaxing Li, Shijun You, Nirit Bernstein, Kailei Tang, Yuanyuan Liu

**Affiliations:** Haixia Institute of Science and Technology, State Key Laboratory of Agricultural and Forestry Biosecurity, College of Life Sciences, Instrumental Analysis Center, Fujian Agriculture and Forestry University, Fuzhou 350002, China; Haixia Institute of Science and Technology, State Key Laboratory of Agricultural and Forestry Biosecurity, College of Life Sciences, Instrumental Analysis Center, Fujian Agriculture and Forestry University, Fuzhou 350002, China; Haixia Institute of Science and Technology, State Key Laboratory of Agricultural and Forestry Biosecurity, College of Life Sciences, Instrumental Analysis Center, Fujian Agriculture and Forestry University, Fuzhou 350002, China; Haixia Institute of Science and Technology, State Key Laboratory of Agricultural and Forestry Biosecurity, College of Life Sciences, Instrumental Analysis Center, Fujian Agriculture and Forestry University, Fuzhou 350002, China; Haixia Institute of Science and Technology, State Key Laboratory of Agricultural and Forestry Biosecurity, College of Life Sciences, Instrumental Analysis Center, Fujian Agriculture and Forestry University, Fuzhou 350002, China; Haixia Institute of Science and Technology, State Key Laboratory of Agricultural and Forestry Biosecurity, College of Life Sciences, Instrumental Analysis Center, Fujian Agriculture and Forestry University, Fuzhou 350002, China; Haixia Institute of Science and Technology, State Key Laboratory of Agricultural and Forestry Biosecurity, College of Life Sciences, Instrumental Analysis Center, Fujian Agriculture and Forestry University, Fuzhou 350002, China; Haixia Institute of Science and Technology, State Key Laboratory of Agricultural and Forestry Biosecurity, College of Life Sciences, Instrumental Analysis Center, Fujian Agriculture and Forestry University, Fuzhou 350002, China; Haixia Institute of Science and Technology, State Key Laboratory of Agricultural and Forestry Biosecurity, College of Life Sciences, Instrumental Analysis Center, Fujian Agriculture and Forestry University, Fuzhou 350002, China; Haixia Institute of Science and Technology, State Key Laboratory of Agricultural and Forestry Biosecurity, College of Life Sciences, Instrumental Analysis Center, Fujian Agriculture and Forestry University, Fuzhou 350002, China; Institute of Soil Water and Environmental Sciences, Volcani Institute, 68 HaMaccabim Road, Rishon LeZion 7505101, Israel; School of Agriculture, Yunnan University, Chenggong District, Kunming 650500, China; Haixia Institute of Science and Technology, State Key Laboratory of Agricultural and Forestry Biosecurity, College of Life Sciences, Instrumental Analysis Center, Fujian Agriculture and Forestry University, Fuzhou 350002, China

## Abstract

*Cannabis sativa* produces a rich array of specialized metabolites, including cannabinoids, terpenoids, and flavonoids, which are of immense therapeutic interest. However, the genetic and regulatory complexity arising from its highly heterozygous genome has obscured a complete understanding of their biosynthesis. Here, we present a fully phased, chromosome-level genome assembly of the wild diploid cannabis accession CSLZ. Comparative genomics and transcriptomics reveal that extensive haplotype-specific variation is a major driver of metabolic diversification within this accession. We find that structural variants and transposable element insertions differentially shape the allelic architecture of key biosynthetic genes, leading to divergent expression and function between haplotypes. This is exemplified by haplotype-specific loss of germacrene D synthase activity, copy number variation in oxidosqualene cyclases, and silencing of a flavonoid glycosyltransferase allele via promoter motif loss. Furthermore, allele-specific expression contributes to the spatiotemporal regulation of metabolic pathways, from cuticle formation in leaves to cannabinoid production in glandular trichomes. Our haplotype-resolved resource uncovers the pervasive role of allelic divergence in generating the specialized metabolome of wild cannabis accession CSLZ, providing a foundation for genomics-guided breeding and metabolic engineering.

## Introduction

Secondary metabolites are pivotal for plant adaptation, defense, and mediating ecological interactions [1]. In the globally significant species *Cannabis sativa* L., these compounds including cannabinoids, terpenoids, and flavonoids define critical agronomic and therapeutic traits, such as chemotype, aroma, and medicinal value [2]. However, elucidating the genetic regulation of these complex pathways is challenging due to the plant’s high heterozygosity and polygenic control. Traditional genomic studies reliant on a single, collapsed reference genome fail to capture the distinct contributions of homologous chromosomes, thereby obscuring haplotype-specific variation that are fundamental to a comprehensive understanding of gene expression and trait heritability.

This challenge is not unique to cannabis but is common among diverse plant lineages, including highly heterozygous diploids and complex polyploids. Recent advances in haplotype-resolved genome assemblies have transformed our ability to dissect allele-specific regulation, uncovering allele-specific expression (ASE), structural variation, and regulatory asymmetry that collectively shape plant traits. For example, allele-aware assemblies identified ∼19% of genes in diploid, hybrid-origin apple [3] displaying ASE during fruit development and diploid potato demonstrating 16.6% of allelic genes exhibiting differential expression and 30.8% differential methylation [4]. In gymnosperms such as *Pinus densiflora*, haplotype-level variation illuminated structural rearrangements and transcription factor expansions driving evolutionary adaptation [5]. Haplotype-resolved assemblies for perennial crops such as lemon (*Citrus limon*) and avocado (*Persea americana*) have clarified the genetic basis of fruit and essential oil traits [6, 7]. The octoploid cultivated strawberry [8] and the tetraploid potato cultivar ‘Otava’ [9] further highlight how phasing massive structural rearrangements in polyploids is key to understanding domestication traits such as fruit color and firmness. In the tea plant (*Camellia sinensis*), a haplotype-resolved genome uncovered extensive structural variation and widespread ASE associated with domestication traits, including dwarfing through selection on brassinosteroid metabolism genes (CsBAS1 and CsDWF4), as well as cold tolerance and herbivore defense [10].

Building on these insights, haplotype-aware genomic frameworks have also advanced our understanding of secondary metabolite biosynthesis, revealing how structural and regulatory divergence between alleles shapes specialized metabolic pathways. For instance, the haplotype-resolved genome of the rubber tree (*Hevea brasiliensis*) revealed that more than 30% of annotated genes exhibiting ASE, indicating an imbalanced genetic contribution to rubber biosynthesis [11]. In perennial plants such as mulberry, resolving haplotypes was crucial for identifying regulatory variation, including promoter indels and missense mutations, that govern anthocyanin accumulation in fruits [12]. In the diploid medicinal plant *Artemisia annua*, a haplotype-aware assembly revealed a positive correlation between artemisinin content and the copy number of the amorpha-4,11-diene synthase (ADS) genes, revealing that gene family expansion is a key factor governing artemisinin yield [13]. In the medicinal autotetraploid crop rhubarb (*Rheum officinale*), the haplotype-resolved genome uncovered numerous allele-differentially-expressed genes, often due to transposable element (TE) insertions in regulatory regions, revealing an imbalanced genetic contribution to the anthraquinone biosynthesis pathway [14]. In vegetatively propagated *Zingiber officinale* (cultivated ginger), haplotype resolution uncovered large inversions linked to infertility, while ASE of key genes such as *C4H* in the phenylpropanoid pathway regulates gingerol biosynthesis [15]. In *C. sinensis*, ASE was further detected in *F3′H* and *CsXDH*, key genes for catechin and caffeine biosynthesis, respectively [10].

Recent progress in cannabis genomics led to a comprehensive *Cannabis* pangenome comprising 181 new and 12 previously published genomes [16]. These resources highlight genomic and structural diversity across hemp, marijuana, and feral or wild accessions, as well as TE activity shaping both the sex chromosomes and the cannabinoid synthase gene clusters. The pangenome revealed that wild Asian accessions maintain distinct genomic resources that differ markedly from domesticated fiber or drug-type varieties, providing valuable allelic and regulatory diversity for understanding secondary metabolism and adaptation. Nevertheless, critical questions remain regarding the haplotype-specific regulatory features that drive allelic imbalance. Despite the growing availability of pangenomes and haplotype-resolved assemblies, the extent to which structural variants (SVs), TEs, and promoter divergence contribute to ASE and metabolic specialization in cannabis remains largely unexplored. In this study, we present a chromosome-scale, haplotype-resolved genome of a wild cannabis accession Linzhi (CSLZ) from Tibet and integrate multi-omics analyses to dissect haplotype-specific regulation of secondary metabolite biosynthesis, including cuticle, terpenoids, cannabinoids, flavonoids, and lignin. Our findings provide new insights into the genetic and regulatory basis of metabolite diversity, trichome development, and adaptation in cannabis, highlighting the critical role of allelic divergence in shaping key agronomic and pharmacological traits.

## Result

### Genome assembly and haplotype chromosome construction

**Figure 1:**
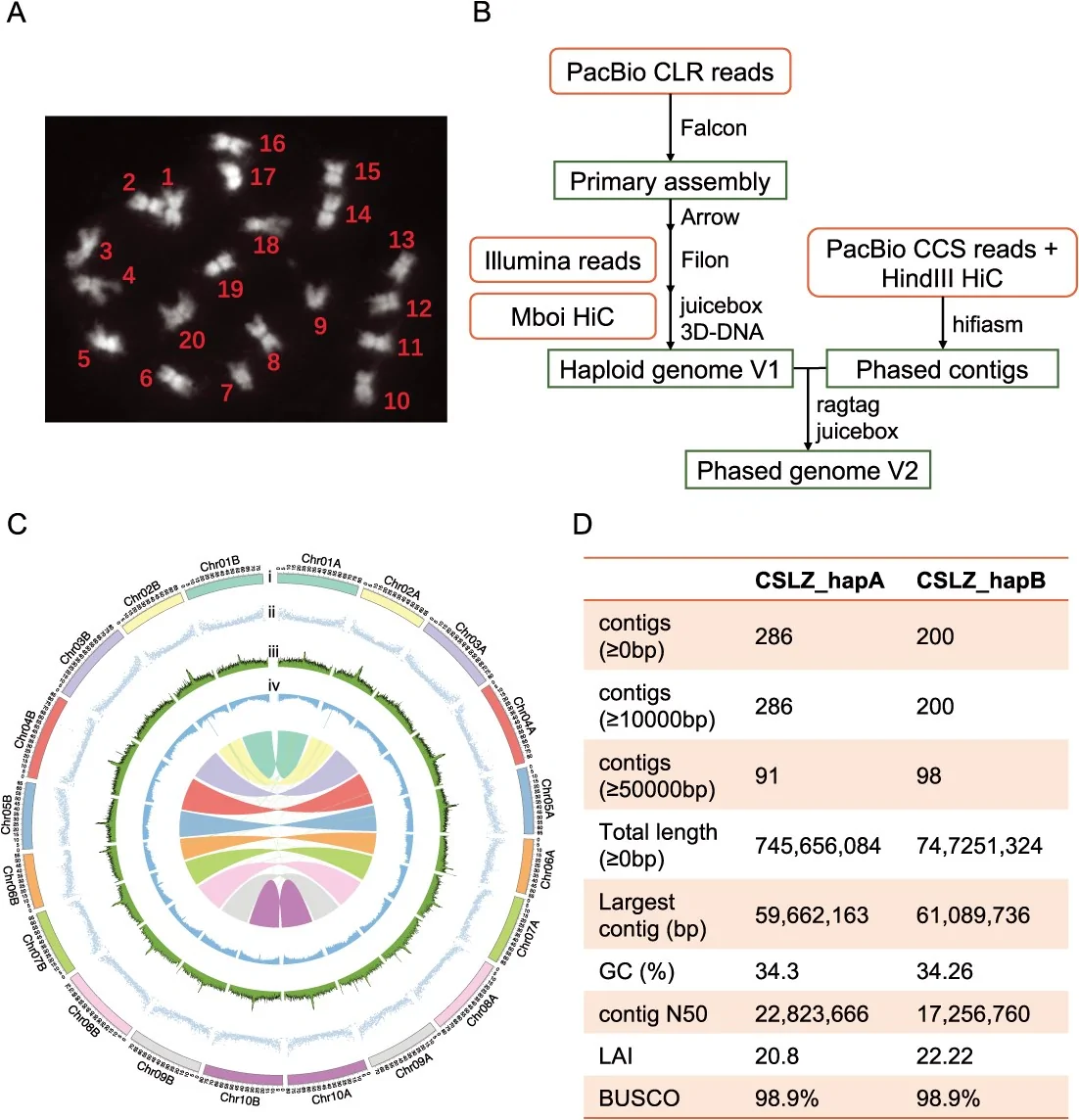
Sequencing samples and genome assembly results. (A) Karyotype of *C. sativa* Linzhi. (B) Workflow of genome assembly. (C) Characteristics of the CSLZ genome. Genomic landscape of the 20 assembled chromosomes. Tracks are displayed from the outer to inner rings as follows: (i) chromosome length (Mb); (ii) gene density; (iii) GC content; and (iv) TE density. All densities were calculated in 100 kb windows. The innermost arcs represent synteny blocks. (D) Summary of genome assembly.

We first performed karyotype analysis to determine the chromosomal composition and ploidy level of the wild cannabis accession CSLZ. The results revealed that CSLZ possesses 10 pairs of chromosomes (2*n* = 20), confirming its diploid status and providing a cytogenetic foundation for subsequent genome assembly (Fig. 1A). To estimate the physical genome size, we performed flow cytometry analysis using *Oryza sativa* ssp. *japonica* as an internal reference, which indicated a genome size of ~777.75 Mb (Fig. S1). We generated a total of 274.39Gb of raw sequences, including 71.04 Gb (94.72×) PacBio CLR reads, 29.25 Gb (39X) PacBio CCS reads, and 70.10 Gb (93×) Illumina paired-end reads with 104 Gb (138.67×) Hi-C data, to construct a chromosome-scale haplotype-resolved assembly. We first assembled haploid genomes and then combined PacBio CCS reads with HindIII Hi-C data to phase contigs, ultimately producing a fully phased, chromosome-level assembly (Fig. 1B). After manual curation of contig order and orientation using Hi-C contact maps, 99.1% of both haplotypes were anchored to 10 chromosomes, with total lengths of 739.64 and 739.45 Mb, respectively (Fig 1C and Fig.  S2), covering ~95.1% of the flow cytometry–estimated genome size. The final assemblies of HapA and HapB totaled 1.48 Gb, with contig N50 values of 22.8 and 17.3 Mb, respectively (Fig. 1D).

The assembly quality was rigorously assessed using multiple complementary metrics. Merqury analysis yielded consensus Quality Value (QV) exceeding 65 for both haplotypes (HapA: 66.03; HapB: 65.60), indicating highly accurate base-level assembly. K-mer completeness was estimated to be >98%, demonstrating that the vast majority of the reliable genomic information was successfully captured. Taxonomic classification using Kraken2 with the Standard-8 database confirmed the absence of viral or nonplant contamination in the final chromosome assemblies (Table S1). In addition, the long terminal repeat (LTR) Assembly Index scores were 20.8 for HapA and 22.2 for HapB, reflecting high continuity and gold-standard assembly quality.

To ensure a comprehensive repeat landscape prior to gene annotation, we integrated multiple complementary approaches, combining standard *de novo* pipelines (RepeatModeler and The Extensive *de novo* TE Annotator) with targeted identification using MITE-Hunter and Tandem Repeats Finder (TRF). Overall, repetitive elements accounted for 70.68% and 72.59% of HapA and HapB, respectively (Table S2). Following repeat masking, we predicted 32 213 and 32 037 protein-coding genes in HapA and HapB, respectively, with both assemblies achieving 98.9% completeness according to BUSCO assessment (Fig. 1D). Compared with previously published cannabis genomes, the CSLZ assembly exhibits higher contiguity and more complete gene representation, underscoring its superior quality and reliability for downstream analyses (Fig. S3).

### Evolutionary insights and gene family expansion in cannabis

**Figure 2:**
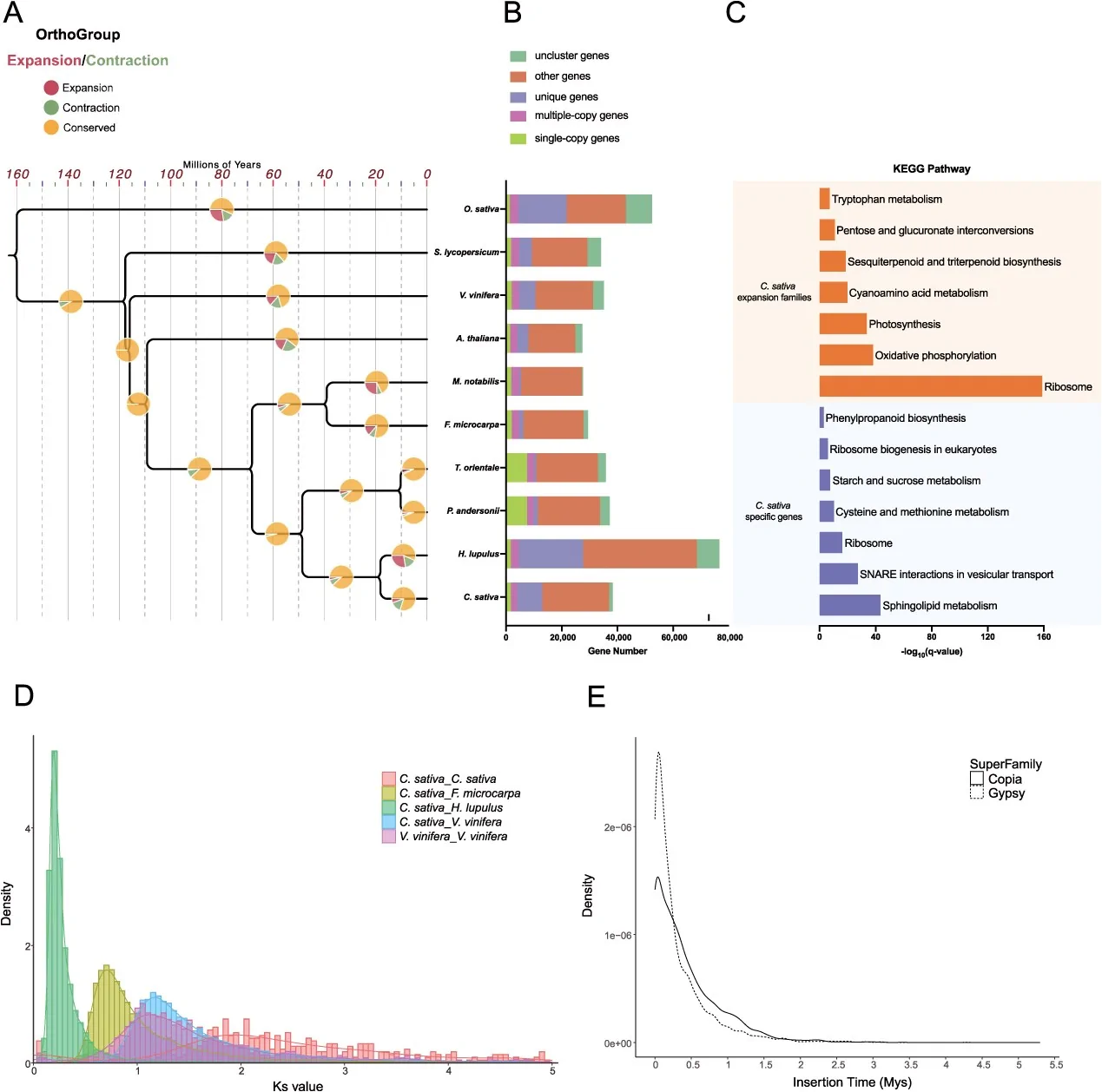
Comparative genomics and TE expansion. (A-B) The phylogenetic tree was constructed based on 21 single-copy genes of *O. sativa*, *S. lycopersicum*, *V. vinifera*, *A. thaliana*, *M. notabilis*, *F. macrocarpa*, *P. andersonii*, *T. orientale*, *H. lupulus*, and *C. sativa*. Orthogroup dynamics, including expansions, contractions, and conservative families, are indicated at each node as defined in the internal figure legend. (C) KEGG enrichment analysis of cannabis expanded gene families (up) and species-specific gene families (down). (D) Ks distributions for paralogs of cannabis and orthologs between cannabis and other species. (E) Insertion time of LTR estimated by sequence divergence of Copia and Gypsy.

Using CSLZ as the representative genome for *C. sativa*, we performed comparative genomics analyses with nine representative plant species spanning key evolutionary nodes: *O. sativa, Solanum lycopersicum, Vitis vinifera, Arabidopsis thaliana, Morus notabilis, Ficus macrocarpa, Parasponia andersonii, Trema orientale*, and *Humulus lupulus*. A total of 606 single-copy orthologous genes were identified and used to construct a phylogenetic tree (Fig. 2A, Table S3). The results showed that *C. sativa* had the closest evolutionary relationship with *H. lupulus*, with an estimated divergence time of 18 (13.2–21.8) million years ago (Mya; Fig. 2A). *P. andersonii* and *T. orientale* divergence in ~10.2 (5.1–15.7) Mya (Fig. 2A). Compared to the most recent common ancestor of *H. lupulus* and *C. sativa*, 686 orthogroups were expanded and 1745 orthogroups contracted in cannabis (Fig. 2A). Kyoto Encyclopedia of Genes and Genomes (KEGG) enrichment analysis revealed that significantly expanded orthogroups were associated with metabolic pathways, including tryptophan metabolism, sesquiterpenoid and triterpenoid biosynthesis, and cyanoamino acid metabolism (Fig. 2C, Table S4). The cyanoamino acid metabolism pathway is a key component of plant defense, contributing to cyanogenic glycoside biosynthesis that deters herbivores and pathogens while influencing defense signaling and broader metabolic regulation [17]. Gene family clustering analysis indicated that 8085 gene families were shared among all 10 species (Table S3), and 4095 genes were unique to *C. sativa* (Fig. 2B, Table S5). KEGG enrichment analysis showed that cannabis-specific genes were primarily enriched in phenylpropanoid biosynthesis, ribosome biogenesis, and starch and sucrose metabolism (Fig. 2C, Table S6). Together, the expanded and cannabis-specific gene families were predominantly associated with the biosynthesis of specialized metabolites such as terpenoids and phenylpropanoids, as well as defense-related pathways including cyanoamino acid metabolism. This coordinated expansion may help explain why the wild accession CSLZ is enriched in medicinal compounds and exhibits strong environmental adaptability.

Whole-genome duplication (WGD) events during cannabis evolution were examined by calculating the density distribution of the synonymous substitution rate (Ks), and two significant peaks were identified (Fig. 2D). The first Ks peak at ~1.98 represented the ancient γ triplication event. The second Ks peak appears at ~0.05, which may represent recent gene expansion. Analysis of LTR expansion revealed that both Copia and Gypsy underwent rapid expansion ~0.1 Aya, suggesting that recent genetic expansion in cannabis may have been driven by intense LTR transposon bursts (Fig. 2E).

### Comparative genomics reveals conserved architecture and lineage-specific innovation in cannabis

**Figure 3:**
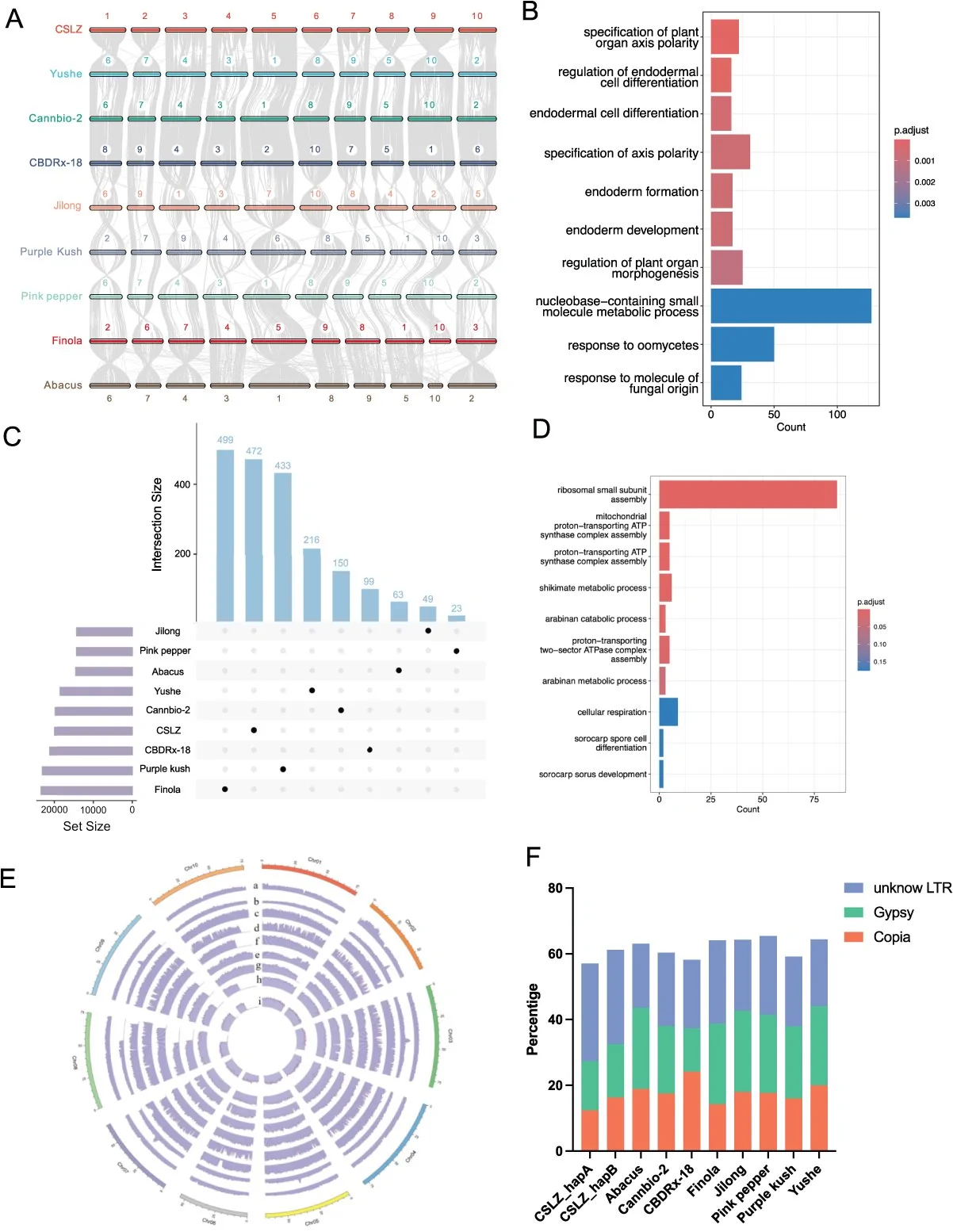
Conservation and specificity among cannabis varieties. (A) Collinearity map of nine cannabis genomes. (B) GO enrichment of genes within conserved regions among cannabis varieties. (C) Unique gene families across cannabis genomes. (D) GO enrichment of unique gene families in CSLZ varieties. (E) Distribution of TEs in the genomes of different cannabis varieties. Tracks are displayed from the outer to inner rings as follows: (a) Linzhi; (b) Abacus; (c) Cannbio-2; (d) CBDRx-18; (e) Jilong; (f) Pink Pepper; (g) Yushe; (h) Finola; (i) Purple Kush. (F) Percentage of different types of TE in the genomes of different cannabis varieties.

To investigate evolutionary divergence among cannabis lineages, we perform whole-genome synteny analysis between CSLZ and other representative cannabis varieties. Overall, chromosomes exhibited clear one-to-one correspondence across varieties, indicating conserved genome collinearity. Nevertheless, numerous structural rearrangements were detected, reflecting substantial intraspecific genomic plasticity (Fig. 3A). Gene ontology (GO) enrichment of 7461 conserved syntenic block genes revealed significant enrichment in terms related to ‘nucleobase-containing small molecule metabolic process’, ‘response to salicylic acid’, and ‘response to oomycetes’, suggesting evolutionary conservation of metabolic and defense functions among cannabis genomes (Fig. 3B, Table S7).

Orthogroup clustering across nine cannabis genomes identified lineage-specific gene families, with the number of variety-specific orthogroups ranging from 23 orthogroups (638 genes) in Pink Pepper to 499 orthogroups (2827 genes) in Finola (Fig. 3C). GO enrichment of CSLZ-specific genes highlighted overrepresentation of categories associated with ribosomal small subunit assembly and ATP synthase complex assembly (Fig. 3D). These processes support enhanced protein synthesis and energy production, implying that CSLZ might have evolved elevated translational and metabolic adaptability. This pattern suggests that, as a wild accession, CSLZ follows an evolutionary trajectory emphasizing both basal cellular function and metabolic flexibility.

We further compared TE content and composition among the nine genomes. Finola harbored the largest TE complement, spanning 711.6 Mb and accounting for 70.5% of its genome (Fig. 3E). Given that LTR retrotransposons comprise the majority of TE sequences, we quantified the relative proportions of Copia, Gypsy, and unclassified LTRs across varieties. In both haplotypes of CSLZ, Copia elements represented the smallest fraction (12.37% and 16.28%, respectively), similar to proportions observed in other cannabis genomes (Fig. 3F, Table S8).

### Transposable element insertions shape allelic regulation of cuticle biosynthesis

**Figure 4:**
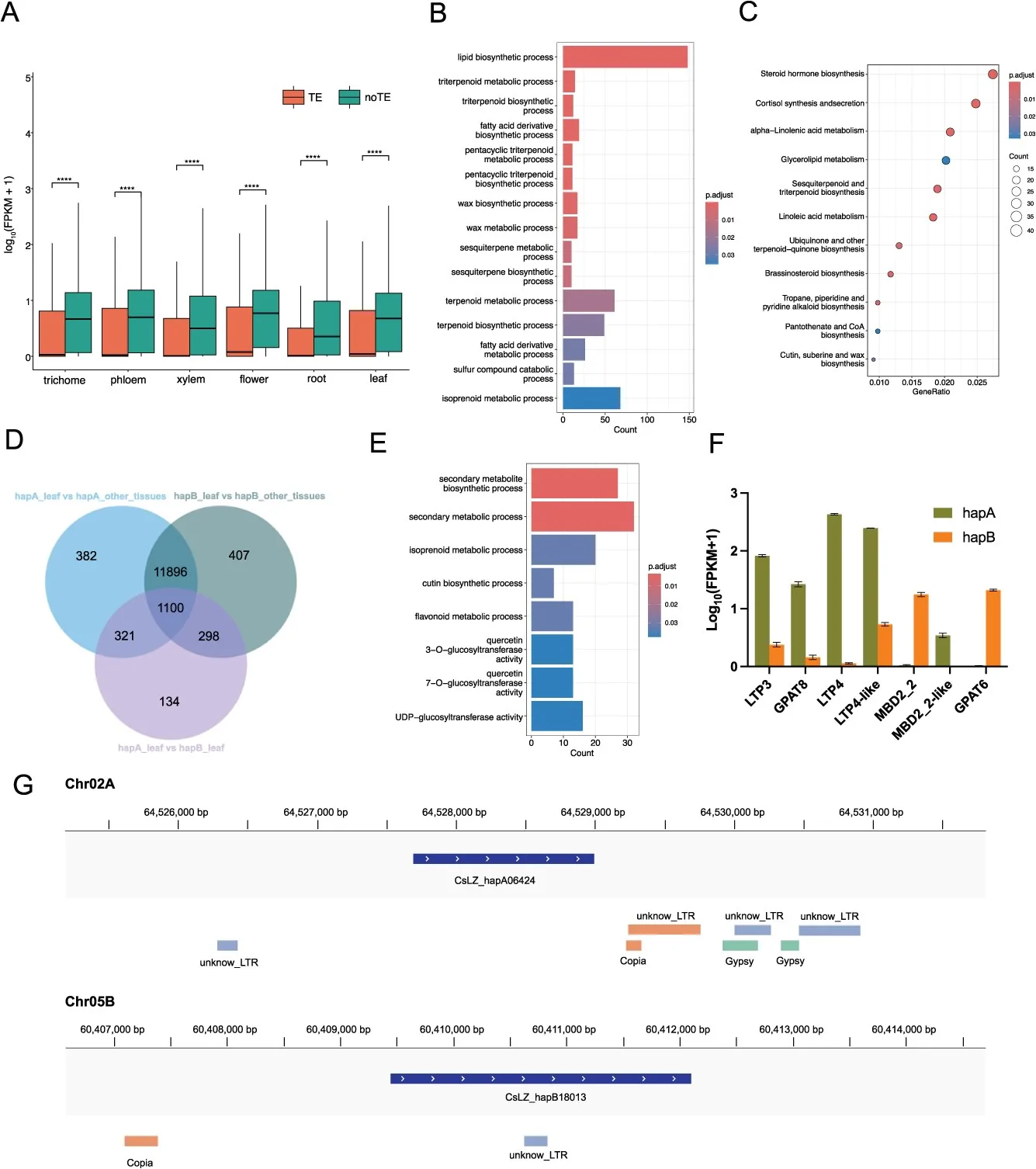
TE-mediated transcriptional suppression and allelic regulation of cuticle biosynthesis (A) Effects of TE insertions on gene expression across different tissues. Statistical significance was determined using the two-sided Mann–Whitney test, **** indicates *P* < 0.0001. (B) GO enrichment analysis of TE-gene. (C) KEGG enrichment analysis of TE-gene. (D) Venn diagrams of the number of different expression genes in the leaf. (E) GO enrichment analysis of leaf tissue-specific genes with allelic differential expression. (F) Expression levels of seven cutin biosynthetic process genes. (G) TE insertion in LTP4-like. LTP4-like_hapA without TE insertion (up) and LTP4-like_hapB with TE insertion (down).

To systematically assess the impact of TEs on gene expression, we classified genes harboring TEs within their genomic regions as TE-associated genes (TE-genes) and the remainder as noTE-genes. Transcriptome sequencing of CSLZ glandular trichomes, female flowers, xylem, phloem, roots, and leaves revealed that noTE-genes consistently exhibited significantly higher expression levels than TE-genes across all tissues (Fig. 4A), indicating a pervasive suppressive effect of TEs on transcriptional activity. GO enrichment analysis of TE-genes showed significant enrichment in pathways related to lipid, terpene, wax, and fatty acid biosynthesis (Fig. 4B, Table S9), while KEGG analysis further highlighted ‘steroid hormone biosynthesis’; ‘sesquiterpenoid and triterpenoid biosynthesis’; and ‘cutin, suberin, and wax biosynthesis’ (Fig. 4C, Table S10). Given that cutin and wax constitute essential components of the leaf cuticle, which mediates protection against desiccation and environmental stress, we focused on leaf transcriptomes to elucidate the molecular basis of cuticle formation.

By comparing gene expression between leaves and other tissues across two haplotypes, and integrating ASE in leaves, we identified 1100 leaf-specific genes exhibiting haplotype-biased expression (Fig. 4D). GO enrichment analysis indicated significant overrepresentation of terms related to secondary metabolic processes, particularly the cutin biosynthetic process (Fig. 4E, Table S11). Seven of these genes encoded enzymes involved in cutin biosynthesis, including three lipid transfer proteins (LTPs), two glycerol-3-phosphate acyltransferases (GPATs), and two HXXXD-type acyltransferase family proteins (Fig. 4F). Notably, LTP genes showed markedly higher expression in haplotype A (Fig. 4F). Structural annotation identified an LTR insertion within the intron of *LTP4-like_hapB* gene, which was absent from *LTP4-like_hapA* (Fig. 4G). These observations suggest that the LTR insertion in LTP4-like_hapB may drive the observed allelic expression divergence, linking TE insertion to regulatory variation in cuticle-associated genes.

### Haplotype-specific structural variation drives diversification of terpene biosynthesis

**Figure 5:**
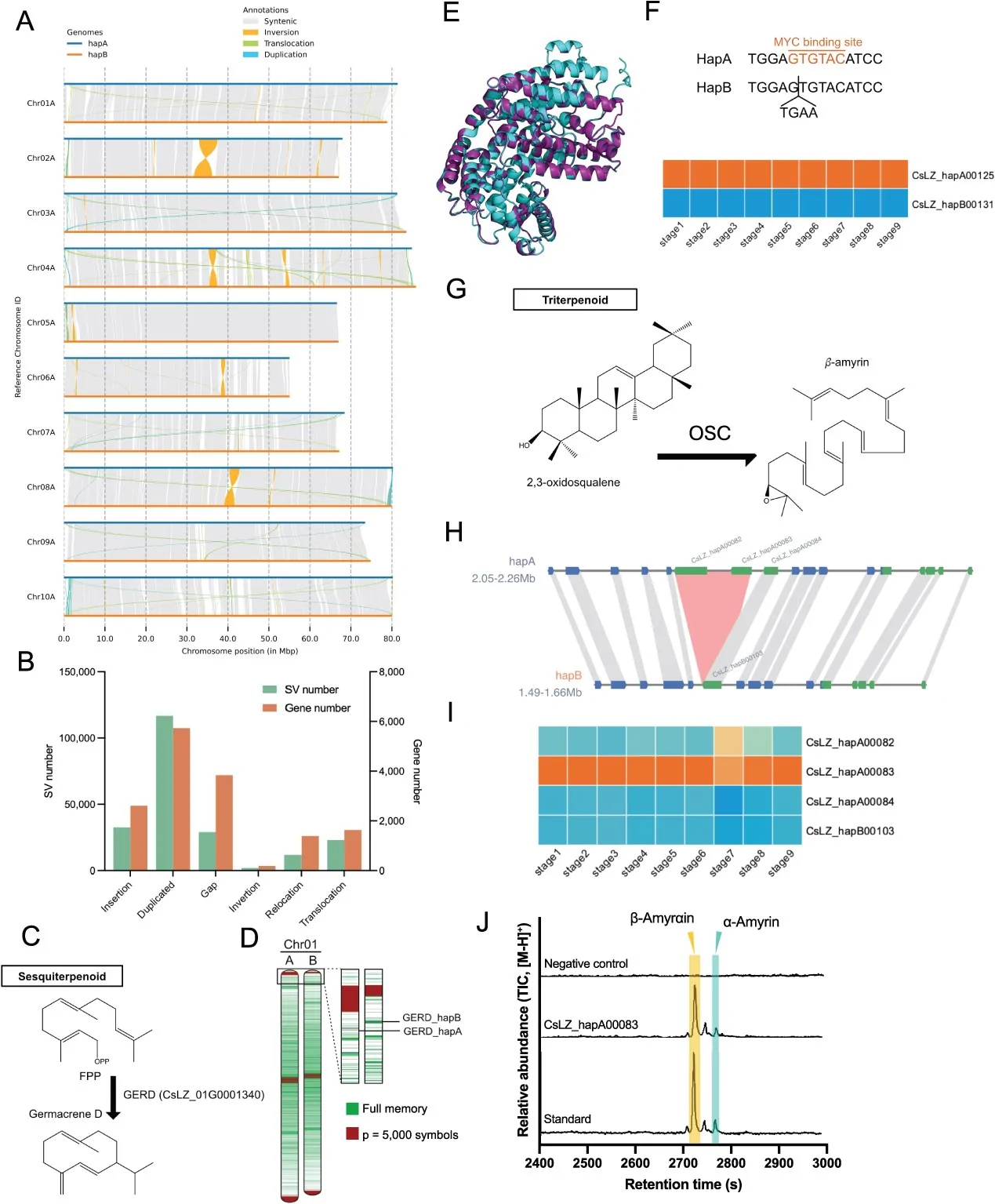
Haplotype-specific structural variation drives allelic divergence in terpene biosynthesis. (A) SVs between HapA and HapB. (B) Total number of variations and overall number of genes containing SVs in the gene region. (C) Structure and catalytic reaction of GERD. (D) Low complexity map of chromosome 1. (E) Multi-sequence comparison between GERD_HapA and GERD_HapB. (F) Differences in GERD expression and promoter region in different haplotypes. (G) Catalytic reaction of OSC. (H) Copy number variation of OSC in different hap. (I) OSC expression heatmap. (J) GC–MS extracted ion chromatogram of m/z 218 in tobacco co-expressing CsLZ_hapA00083 and CsHMGR1.

Genomic variation is a major driver of gene innovation and functional diversification. To investigate haplotype-specific SVs, we aligned HapB against HapA using MUMmer and identified SVs with SyRI (Fig. 5A). We detected 32 594 insertion, 116 866 duplicated sequences, 29 055 gap, 1966 inversion, 11 790 relocation, and 22 989 translocation (Fig. 5B, Table S12), indicating extensive structural asymmetry between haplotypes. To further characterize the genomic architecture underlying these varians, we profiled genome-wide sequence complexity, revealing the distribution of low-complexity regions (LCRs) across all chromosomes (Fig. S4).

In cannabis, terpenoids play pivotal ecological roles, mediating plant–insect–microbe interactions and contributing to chemotypic and pharmacological diversity [18]. Sesquiterpenes determine characteristic aroma profiles and synergize with cannabinoids, whereas triterpenes underpin bioactive compound diversity [19]. Germacrene D, a sesquiterpene with antibacterial and anti-inflammatory activity, is produced from farnesyl diphosphate by germacrene-D synthase (GERD; Fig. 5C) [20]. Genomic complexity analysis using AlcoR revealed that the HapB allele resides within a low-complexity region, in contrast to the high-complexity context of the HapA allele (Fig. 5D). To further characterize the structural environment of this locus, we performed a detailed synteny analysis of the local chromosomal region (Figs S5 and S6). We identified a haplotype-specific structural variant at the GERD locus; the HapB allele lacks the 5′ region encoding the first 14 amino acids, likely abolishing enzymatic function (Figs 5E and S7). Moreover, a four-base insertion in the GERD_HapB promoter disrupts an MYC transcription factor-binding motif present in GERD_HapA (Fig. 5F). Transcriptomic profiling across nine female flower developmental stages revealed strong expression of GERD_HapA, whereas GERD_HapB was transcriptionally inactive (Fig. 5F). These findings suggest that haplotype-specific structural and regulatory variations jointly drive allelic expression divergence of a key terpene synthase (TPS).

β-Amyrin, a triterpenoid alcohol that serves as a core precursor for diverse bioactive triterpenes, is synthesized by 2,3-oxidosqualene cyclases (OSCs) that catalyze the cyclization of 2,3-oxidosqualene into α- and β-amyrin (Fig. 5G) [21]. The OSC gene family exhibits haplotype-specific copy number variation in CSLZ, with three copies in HapA and one in HapB (Fig. 5H). Expression profiling across flower development revealed that a single HapA copy (CsLZ_hapA00083) is highly expressed throughout all stages, while the HapB copy remains silent (Fig. 5I). Functional characterization by co-expression of CsLZ_hapA00083 with 3-Hydroxy-3-methylglutaryl CoA reductase (HMGR) in *Nicotiana benthamiana* confirmed β-amyrin production (0.347 μg/ml; Fig. 5J). Collectively, these results indicate that gene duplication and subsequent expression divergence in HapA created an active, three-copy expansion of OSC, while HapB retained a single, silent copy, revealing haplotype-specific innovation in triterpenoid biosynthesis.

### Allelic regulation of flavonoid biosynthesis in glandular trichomes

**Figure 6:**
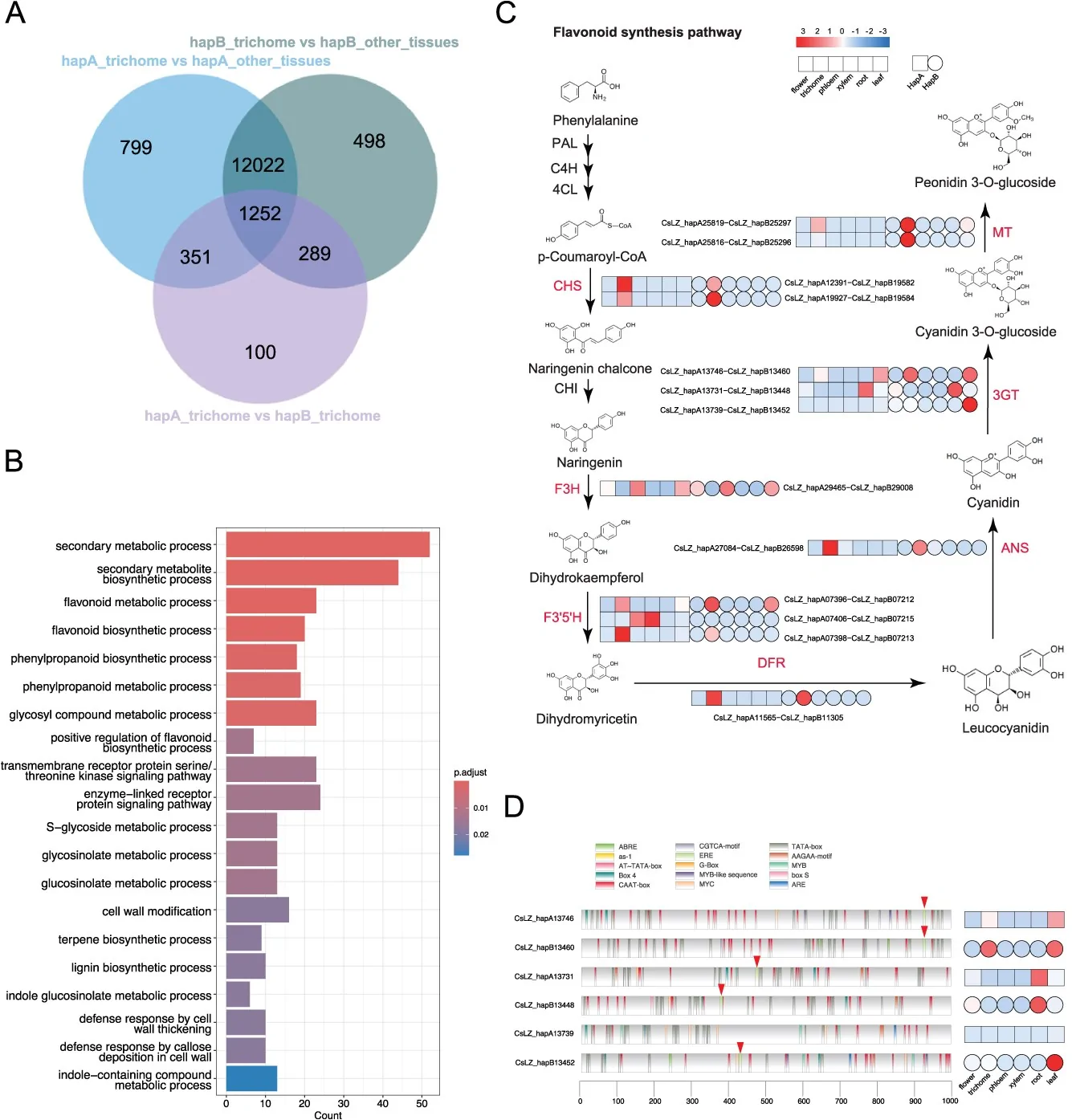
Haplotype-dependent transcriptional regulation of flavonoid biosynthesis in glandular trichomes. (A) Venn diagrams of the number of different expression genes in the glandular trichome. (B) GO enrichment analysis of glandular trichome tissue-specific genes with allelic differential expression. (C) Heatmap of flavonoid synthesis pathway gene expression in different tissues. (D) Transcription factor binding sites and gene expression levels of six 3GT haplotypes. The triangle represents the ERE motif.

To examine the contribution of ASE to tissue-specific secondary metabolism, we performed transcriptome sequencing across six CSLZ representative tissues of *C. sativa*: roots, stem xylem, stem phloem, leaves, female flowers (without trichomes), and glandular trichomes. Correlation analysis revealed that global expression profiles were primarily shaped by tissue identity, roots formed a distinct cluster from aerial organs, glandular trichomes clustered closely with female flowers, and leaves grouped with stem phloem (Fig. S8). To identify genes subject to both allelic and spatial regulation, we defined a set of 1252 genes specifically expressed in glandular trichome and differentially expressed between the two haplotypes (Fig. 6A). GO enrichment analysis revealed that these genes were significantly enriched for terms associated with secondary metabolic processes, particularly the biosynthesis of flavonoids and phenylpropanoids (Fig. 6B, Table S13).

Flavonoids, key derivatives of the phenylpropanoid pathway, serve multifaceted roles in cannabis, contributing to ultraviolet (UV) protection, pigmentation, and defense against pathogens and oxidative stress [22, 23]. To assess allelic contributions to trichome-specific flavonoid biosynthesis, we analyzed ASE within the flavonoid biosynthetic pathway. Many genes specifically expressed in glandular trichome also exhibited strong ASE bias, suggesting coordinated genetic and spatial regulation (Fig. 6C). For example, the flavonoid 3′,5′-hydroxylase (F3′5′H), which catalyzes the hydroxylation of dihydrokaempferol to dihydromyricetin, showed pronounced haplotype-specific expression, four haplotypes were predominantly expressed in glandular trichomes, one was mainly active in stem xylem and phloem, and one was not transcribed. Both copies of *O*-methyltransferase (MT) were preferentially expressed in glandular trichomes, with higher expression from the hapB allele. 3-O-glycosyltransferase (3GT), which glycosylates cyanidin to cyanidin 3-O-glucoside, showed marked allelic asymmetry. Among six 3GT haplotypes, most were expressed in glandular trichomes, roots, and leaves, whereas one hapA copy (CsLZ_hapA13739) was transcriptionally inactive across all tissues. Comparative promoter analysis revealed that the non-expressed CsLZ_hapA13739 promoter lacked an ethylene-responsive element motif conserved in transcriptionally active 3GT promoters of other species (Fig. 6D), such as *Vaccinium corymbosum* and *V. vinifera* [24, 25]. The loss of this regulatory motif likely contributes to transcriptional silencing of CsLZ_hapA13739, suggesting that promoter motif variation mediates allelic regulation of flavonoid biosynthesis and contributes to tissue-specific metabolic diversification in cannabis.

### Haplotype-resolved dynamics of gene expression during female flower development

**Figure 7:**
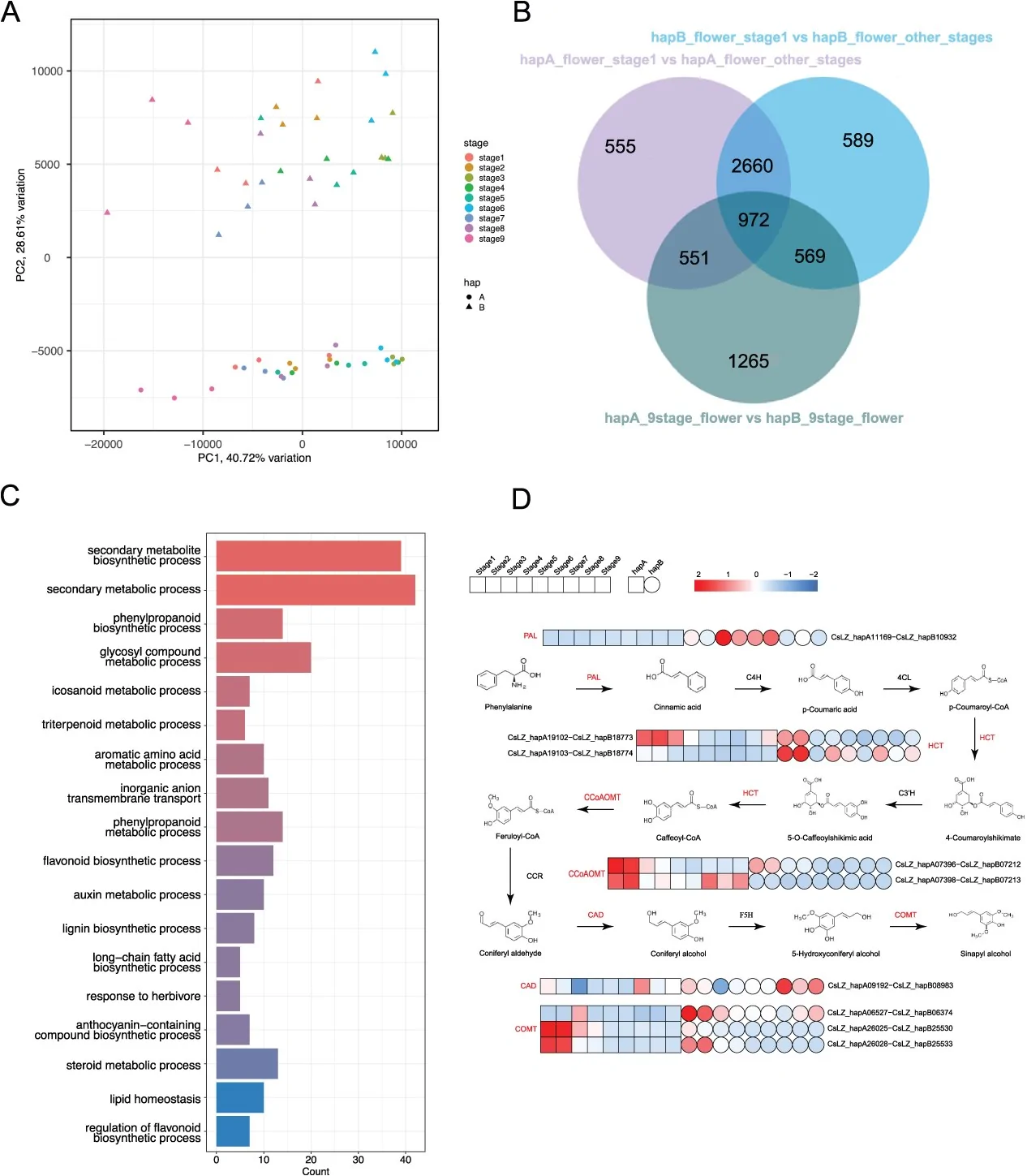
Allele-specific regulation and developmental control of secondary metabolism in female flowers. (A) PCA of the female flower RNA-seq data. (B) Venn diagrams of the number of different expression genes in female flower. (C) GO enrichment analysis of differentially expressed genes with allelic differential expression during female flower development. (D) Heatmap of phenylpropane biosynthesis pathway gene expression during female flower development.

The female cannabis flower is characterized by a perianth enclosing an ovule, protective bracts that host the highest density of glandular trichomes, and two stigmas. From week 1 to week 9 of flowering, calyx size increases from 1 to 6 mm, accompanied by a marked rise in trichome density [26, 27]. To investigate the contribution of ASE to female flower development and metabolism, we performed transcriptome sequencing across nine flowering stages of the CSLZ accession. Principal component analysis (PCA) revealed that developmental stage accounted for the largest proportion of transcriptional variance (PC1, 40.72%), while haplotype identity contributed a secondary but distinct axis (PC2, 28.61%; Fig. 7A). This indicates that both developmental progression and genetic architecture shape the global transcriptional landscape during cannabis female flower development.

We identified 972 differentially expressed alleles that were responsive to both factors, showing variation between haplotypes at the same developmental stage and across stages within the same haplotype. (Fig. 7B). GO enrichment of this intersecting set revealed overrepresentation of genes involved in secondary metabolic processes, including phenylpropanoid, triterpenoid, flavonoid, and lignin biosynthesis (Fig. 7C, Table S14). Within lignin biosynthetic pathway, phenylalanine ammonia-lyase (PAL) was predominantly expressed from HapB during early and middle developmental stages, while hydroxycinnamoyl-CoA shikimate/quinate hydroxycinnamoyltransferase (HCT), caffeoyl-CoA MT, cinnamyl alcohol dehydrogenase and caffeic acid MT genes displayed haplotype-biased expression across all the stages (Fig. 7D). Promoter analysis of the non-expressed allele HCT2_HapA (CsLZ_hapA19103) revealed deletion of a Box 4 light-responsive *cis*-element due to a haplotype-specific base substitution (Fig. S9), likely causing the observed ASE.

### Allelic regulation of trichome development and cannabinoid biosynthesis

**Figure 8:**
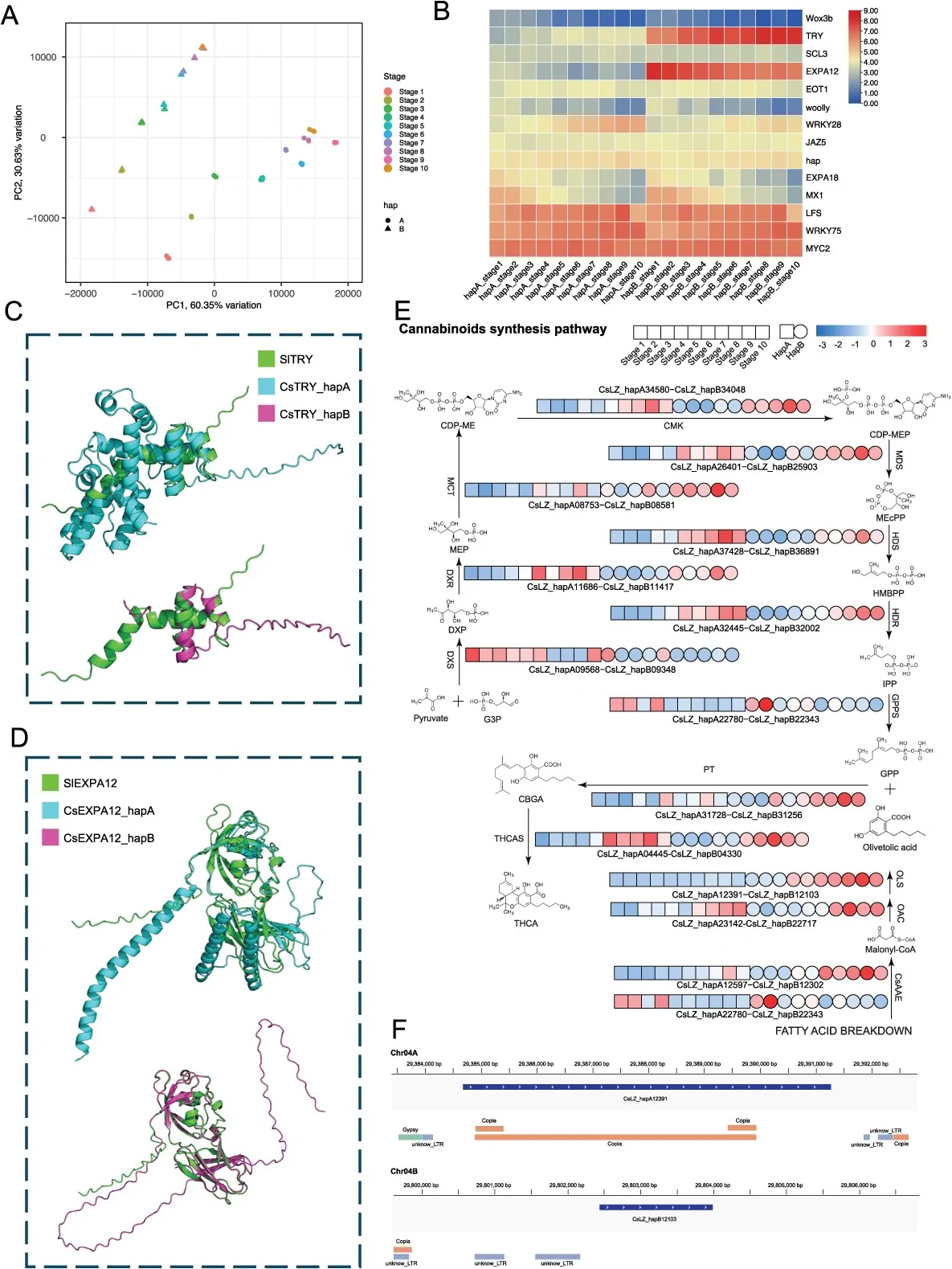
Allele-specific regulation and structural divergence shape trichome development and cannabinoid biosynthesis. (A) PCA of the glandular trichomes RNA-seq data. (B) Heatmap of expression levels of cannabis homologs of tomato trichome development regulators. (C) Protein structure of SlTRY, CsTRY_hapA, and CsTRY_hapB. (D) Protein structure of SlEXPA12, CsEXPA12_hapA, and CsEXPA12_hapB. (E) Heatmap of cannabinoid biosynthesis pathway gene expression during glandular trichome development. (F) TE insertion in OLS. OLS_hapA with TE insertion (up) and OLS_hapB without TE insertion (down).

In *C. sativa*, glandular trichomes are the primary sites for the biosynthesis and storage of pharmaceutically valuable secondary metabolites, including cannabinoids and terpenes, which collectively determine the plant’s medicinal potency. To dissect the allele-specific regulatory basis of trichome development and metabolism, we conducted allele-resolved RNA sequencing across nine developmental stages of glandular trichomes. PCA of the transcriptome data revealed that haplotype identity was the major determinant of expression variance, driving separation along the diagonal of the first principal component (Fig. 8A).

Examination of cannabis homologues of known glandular trichome regulators in tomato uncovered dynamic expression patterns during trichome ontogeny (Fig. 8B, Table S15). Among these, the negative regulator TRIPTYCHON (TRY) and the positive regulator EXPANSIN 12 (EXPA12) exhibited striking haplotype-specific transcriptional bias (Fig. 8B). *TRY_HapB* was strongly expressed, whereas *TRY_HapA* expression was nearly undetectable; a similar trend was observed for *EXPA12*, with *EXPA12_HapB* highly expressed and *EXPA12_HapA* transcriptionally silent (Fig. 8B).

Structural comparison of the TRY locus revealed extensive haplotype divergence. In tomato, TRY encodes a 94-amino-acid protein, whereas the actively transcribed cannabis TRY_HapB produces a 78-amino-acid protein and the inactive TRY_HapA encodes a 239-amino-acid protein (Fig. 8C). Predicted tertiary structures of the two cannabis alleles are distinct, with TRY_HapB more closely resembling the tomato counterpart (Fig. 8C), suggesting that TRY_HapB may have acquired lineage-specific functions in regulating glandular trichome density and spatial patterning. *EXPA12* belongs to the expansin family, which is broadly conserved among dicotyledonous plants and mediates cell wall loosening by disrupting hydrogen bonds between polysaccharides, thereby facilitating cell expansion under acidic conditions [28]. Canonical expansin protein comprises both an expansin C-terminal domain and a lytic transglycolase domain. Actively transcribed EXPA12_HapB retains both domains, consistent with full functional potential, whereas the non-expressed EXPA12_HapA lacks the C-terminal domain required for full activity (Fig. 8D and Fig.  S10). This combination of structural truncation and haplotype-specific expression indicates functional divergence between alleles, which may influence the morphological and developmental specialization of cannabis glandular trichomes.

Given that cannabinoids are the principal bioactive compounds in cannabis, we next profiled the transcriptional dynamics of the cannabinoid biosynthetic pathway during trichome development. Gene expression heatmaps revealed stage-dependent activation of key biosynthetic enzymes and widespread allelic expression divergence (Fig. 8E). 1-Deoxy d-xylulose-5-phosphate synthase and acyl-activating enzyme were preferentially expressed during early stages, whereas most other pathway genes peaked at later stages. Notably, all genes except olivetol synthase (OLS) were expressed from both haplotypes. OLS, which catalyzes the formation of olivetolic acid, a precursor of cannabigerolic acid, showed extreme allelic bias, with OLS-HapB highly expressed in late developmental stages (stage 9), while OLS-HapA remained almost silent. Structural analysis revealed three consecutive TE insertions in the intron of *OLS-HapA* (Fig. 8F), leading to substantial structural divergence relative to OLS-HapB. These TE insertions likely contribute to observed allelic silencing, suggesting how TE-induced SVs can rewire allelic regulation and shape metabolic specialization in cannabis glandular trichomes.

## Discussion

The immense therapeutic and ecological significance of specialized metabolites in plants, such as the complex cannabinoid, terpenoid, and flavonoid profiles of *C. sativa*, demands a complete understanding of their regulatory control. Although the key biosynthetic pathway genes have been identified, the genetic and regulatory mechanisms governing their production remain incompletely understood, particularly in the context of the plant’s highly heterozygous and repetitive genome. To address this gap, we generated a high-quality, fully phased diploid genome of the wild cannabis accession CSLZ, enabling an allele-aware exploration of genomic evolution and metabolic specialization. Integrative transcriptomic and structural analyses reveal extensive structural and regulatory asymmetry between homologous chromosomes, demonstrating that haplotype-specific variation is a critical, yet underappreciated, driver of metabolic divergence in this wild accession, directly shaping specialized metabolite biosynthesis and tissue-specific accumulation.

Our comparative genomic analyses revealed that *C. sativa* has experienced lineage-specific expansion of gene families involved in secondary metabolism and primary-metabolite support pathways. Expanded orthogroups include those linked to tryptophan, sesquiterpenoid, and triterpenoid biosynthesis, consistent with the amplification of TPS and cytochrome P450 families observed in other metabolite-rich species such as *Pinaceae* [5] and *R. officinale* [14]. These expansions likely enhance both metabolic capacity and environmental resilience, coupling biosynthetic innovation with stress adaptation. Enrichment of cannabis-specific gene families in phenylpropanoid biosynthesis, ribosome biogenesis, and starch and sucrose metabolism further suggests that elevated primary metabolic flux supports intensified secondary metabolism in specialized tissues such as glandular trichomes.

A central finding of this study is the pervasive role of TEs in shaping allelic expression and functional divergence, particularly within specialized metabolic pathways. Genes harboring TEs within their structure or regulatory regions consistently exhibited significantly reduced expression across all sampled tissues. This global suppressive effect likely arises from multiple mechanisms, including the disruption of gene structure, altered DNA methylation, chromatin remodeling, or the introduction of novel *cis*-regulatory elements, such as transcription factor binding sites [29]. For example, TEs modulate gene expression by influencing transcription factor binding in the cotton genus [30]. In *Brassica*, TEs reshape local expression landscapes through methylation and structural variation [31]. Similar observations were reported in *P. densiflora* [5], where LTR retrotransposons affect resin biosynthetic genes, and modulate DNA methylation and expression of vanillin biosynthetic genes in *Vanilla planifolia* [32]. In CSLZ, TE-associated genes were enriched in lipid, terpene, and wax biosynthetic pathways that contribute to cuticle formation and environmental adaptation. Notably, an LTR insertion in the intron of LTP4-like gene of haplotype B coincided with transcriptional repression, while three consecutive TE insertions in the intron of *OLS-HapA* likely resulted in near-complete transcriptional silencing of the ‘allele’. These findings are consistent with evidence from *Arabidopsis* and tomato showing that intronic regions, being longer and more permissive, frequently accommodate TE insertions that modulate gene regulation [33]. Collectively, our results highlight TEs as dynamic agents of allelic regulatory divergence and metabolic innovation in ‘cannabis’. Through both disruptive and *cis*-regulatory effects, recent TE activity has rewired expression landscapes in a haplotype-specific manner, fostering transcriptional plasticity and chemotypic diversity across tissues and developmental stages.

The cannabis genome exhibits widespread structural variation, with numerous inversions, translocations, and duplications differentiating the two haplotypes. Many of these directly impact genes encoding key metabolic enzymes, such as GERD and OSC. At the *GERD* locus, the HapB allele exhibits both a truncation and the disruption of an MYC-binding motif in its promoter, which abolish its expression and leave the HapA allele as the sole active copy. This finding is consistent with evidence from *Taraxacum kok-saghyz* (Russian dandelion), where the transcription factor TksMYC2 binds to the promoter of the germacrene A oxidase gene via a homologous motif, and overexpression of TksMYC2 enhances germacrene A oxidase expression in tobacco [34]. Given that germacrene A and germacrene D are structural isomers, it is plausible that the promoter mutation in GERD-HapB disrupts MYC transcription factor binding, thereby leading to the loss of its expression. Furthermore, haplotype-specific expansion of *OSC* in *HapA* contributes to differential triterpenoid biosynthesis, suggesting that tandem duplication followed by asymmetric retention and regulatory remodeling drives chemical diversification. The similar patterns were observed in *P. densiflora*, where large-scale SVs underlie allelic diversity in TPS and phenylpropanoid gene families, facilitating differential accumulation of resin acids and volatiles across tissues [5]. Likewise, expansion of ADS correlates with enhanced artemisinin content in *A. annua* [13]. Collectively, these patterns highlight structural rearrangements as an important mechanism linking haplotype divergence to metabolic specialization.

At the regulatory level, *cis*-element variation shapes allelic expression landscapes and contributes to tissue-specific metabolic specialization. In cannabis, promoter polymorphisms appear to account for allele-specific transcriptional differences through the loss of key regulatory motifs. For example, transcriptional inactivation of one 3GT allele coincides with the loss of ERE/GCC-box motifs, which are well-known positive *cis*-acting elements enhancing transcription in *Arabidopsis* [35, 36] and other dicots [37]. Similarly, an HCT allele is inactivated due to the absence of a light-responsive Box4 element, which is otherwise conserved in the phenylpropanoid gene promoters of *S. tuberosum* [38] and *Agrostis stolonifera* [39]. This is consistent with the high-frequency variation observed in the promoter regions of ASE genes associated with hybrid vigor in maize parents [40], providing new perspectives for investigating how promoter region variation between alleles influences gene expression.

Integration of haplotype-resolved and tissue-specific transcriptomes revealed that ASE is tightly aligned with spatial metabolic partitioning. For example, glandular trichomes preferentially express HapB alleles of trichome regulators, including CsTRY and CsEXPA12, which are predicted to produce functional proteins. In contrast, the corresponding HapA alleles encode structurally divergent proteins, CsTRY-HapA encodes a protein with a distinct predicted tertiary structure, and CsEXPA12-HapA lacks the C-terminal expansin domain required for full activity. Similarly, metabolic polarization is observed for pathway genes, with OLS-HapB driving cannabinoid biosynthesis in trichomes, whereas HapA alleles dominate phenylpropanoid and lignin pathways in structural tissues. This developmental and haplotype-specific partitioning likely represents an adaptive strategy to balance growth, defense, and reproduction, paralleling observations in *V. planifolia*, where phased alleles and gene dosage shape vanillin and phenolic compound biosynthesis [32]. In potato, haplotype-level analyses of starch and steroidal glycoalkaloid (SGA) pathways identified 2169 haplotypes across 119 genes, ~70% of which are transcriptionally active in a tissue-specific manner. Structural variation among these haplotypes contributes to differential expression and metabolic partitioning between the biosynthesis of starch and SGA. Together, these findings illustrate how haplotype diversity, transcriptional specialization, and metabolic partitioning collectively shape phenotypic diversification and functional adaptation in plants [41].

The pervasive structural and regulatory asymmetry observed between the two CSLZ haplotypes provides valuable insight into the evolutionary forces shaping the cannabis genome. The maintenance of such extensive allelic divergence, ranging from presence/absence variation to ASE, suggests that balancing selection may play a key role in preserving genetic diversity in wild populations [42]. By maintaining two functionally distinct haplotypes, wild cannabis potentially benefits from a broader repertoire of biosynthetic and defense capabilities, a genomic architecture that underpins heterosis (hybrid vigor) [43]. For example, divergent alleles of resistance genes or metabolic enzymes allow the plant to respond to a wider array of environmental stresses. This landscape contrasts with domesticated varieties, where intense artificial selection for specific traits (e.g. high THC or fiber yield) has likely driven the fixation of favorable alleles and reduced heterozygosity in target regions [44, 45]. Whereas domestication favors genetic uniformity, our data suggest that wild populations rely on ‘allelic plasticity’, the dynamic regulation of divergent alleles—to cope with fluctuating environments. Thus, the haplotype-resolved asymmetry uncovered here represents an ancestral adaptive strategy that may have been partially diminished or reshaped during the breeding of modern cultivars [45].

Together, these findings establish a coherent framework linking haplotype-specific genome architecture, structural and regulatory divergence, and specialized metabolism in wild cannabis. The allelic perspective uncovered here reveals how genome phasing can illuminate hidden layers of regulation underlying metabolic diversity. Beyond evolutionary insights, this framework provides a foundation for precision breeding and metabolic engineering, which enables targeted manipulation of favorable haplotypes to optimize cannabinoid and terpene production for medicinal and industrial applications.

## Materials and methods

### Plant material and growth conditions

The CSLZ plants used for PacBio sequencing, RNA-seq, and *de novo* genome assembly were propagated and grown from cuttings. Cuttings from mother plants collected from Linzhi County, Tibet, with an average altitude of 3100 m (94.397312° E, 29.617220° N). Cuttings were rooted in the controlled chamber at 24 ± 2°C with a light–dark cycle of 18/6 h, at 60% relative humidity (RH) under white light at an intensity of 140 μmol m^−2^ s^−1^. After rooting, plantlets were transplanted to a 2.4 l pot growing under the same cultivation conditions. Each vegetative plant was placed in an individual pot and watered daily with 500 ml of a nutrient solution with an N-P-K ratio of 3:1:1; pH 6.8 ± 0.1. To initiate flowering, the 6-month-old plants were transferred to a peat–perlite–vermiculite medium [5:1:1 (v/v/v)] in 7.6 l pots and grown at 24 ± 2 °C with a light/dark cycle of 12/12 h, at 60% RH under white light at an intensity of 140 μmol m^−2^ s^−1^ in the controlled chamber. Each plant was watered daily with 500 ml of a nutrient solution with an N-P-K ratio of 2:1:5; pH 6.8 ± 0.1. Genomic DNA was extracted from fresh young leaves for genome sequencing; mixed samples of aboveground tissues (stems, buds, leaves, flowers), and individual samples of female flowers and glandular trichomes were collected for ISO-seq for genome assembly and gene annotation. The roots, stem xylem, stem phloem, leaves, glandular trichomes, and female flowers without trichomes were collected for RNA-seq to compare gene expression differences in different tissues. Female flowers (1–9 weeks after flowering) and glandular trichomes (1–10 weeks after flowering) were collected for RNA-seq to compare gene expression dynamics during their development.

### DNA sequencing

Genome DNA (gDNA) was extracted from young leaf tissues of CSLZ samples using the CTAB method. DNA quality and quantity were assessed using three approaches: (i) NanoDrop 2000 spectrophotometer (Thermo Fisher Scientific), (ii) agarose gel electrophoresis, and (iii) Qubit fluorometer (Invitrogen). The extracted DNA was then purified with AMPure PB beads (PacBio, 100-265-900), yielding high-quality gDNA with concentrations ≥100 ng/μl and total amounts ≥10 μg for subsequent library construction.

Total RNA was extracted from mixed aboveground tissues and individual samples of female flowers and glandular trichomes using the TRIzol method. RNA integrity, concentration, and quantity were assessed using Thermo Fischer Scientific and Agilent 2100 Bioanalyzer (Agilent Technologies), resulting in high-quality RNA with concentrations ≥300 ng/μl and total amounts ≥2 μg for downstream library preparation.

For whole-genome resequencing, DNA libraries were constructed with an insert size of 300–400 bp and sequenced on the Illumina NovaSeq 6000 platform. Low-quality reads, adapter contamination, and PCR duplicates were removed, yielding ~70.10 Gb of clean data (~93× coverage) from a total raw sequencing volume of ~71.92 Gb.

High-molecular-weight genomic DNA for PacBio sequencing was extracted from fresh young leaf tissues of a single plant using the DNeasy Plant Mini Kit. Fragments >15 kb were selected using the BluePippin system, and SMRTbell libraries (30–50 kb) were constructed following PacBio protocols. Sequencing on the PacBio Sequel II platform generated 71.04 Gb of data (~94.7× coverage). Additionally, a separate PacBio SMRTbell library was prepared using the SMRTbell® Express Template Prep Kit 2.0 (PacBio, PN 101-853-100) and sequenced on a single Sequel II cell, yielding 29.5 Gb (~39× coverage).

Hi-C libraries were prepared from young leaf tissues of the same individual using an *in situ* protocol. Briefly, chromatin was cross-linked with formaldehyde, lysed, and digested with the six-base restriction enzyme HindIII. The resulting DNA ends were filled, biotin-labeled, and ligated. Cross-links were reversed, and DNA was sheared by sonication. Biotin-enriched fragments were captured to construct Hi-C libraries, which were subsequently sequenced on the Illumina NovaSeq 6000 platform.

### RNA sequencing and experimental design

To ensure statistical robustness, the experiment was conducted using a randomized complete block design. Three independent biological replicates were collected for each tissue type (female flowers, glandular trichomes, and other tissues) at the specified developmental stages. No sample pooling was performed to preserve biological variance.

After passing quality assessment of independent RNA samples from female flowers and glandular hairs, eukaryotic mRNA was enriched using Oligo(dT)-conjugated magnetic beads. The mRNA was then fragmented, and first-strand cDNA was synthesized using hexamer random primers. Second-strand cDNA synthesis was performed by adding buffer, dNTPs, DNA polymerase I, and RNase H. The resulting double-stranded cDNA was purified using AMPure XP beads. Purified cDNA was subsequently end-repaired, A-tailed, and ligated to sequencing adapters, followed by fragment size selection with AMPure XP beads. PCR amplification was performed, and the amplified products were purified with AMPure XP beads to generate the final RNA-seq library. Library quantification was first performed using Qubit 2.0, and fragment size distribution was assessed with the Agilent 2100 Bioanalyzer. After confirming expected insert sizes, library concentration was accurately determined by qPCR to ensure sequencing quality. Paired-end sequencing was then performed on the Illumina HiSeq 2500 platform.

For PacBio Iso-Seq, mixed RNA samples from aboveground tissues (stem, bud, leaf, flower) were used to construct SMRTbell libraries with the SMRTbell® Express Template Prep Kit 2.0 (Pacific Biosciences, PN 101-853-100). The procedure was as follows: (i) reverse transcription using Oligo(dT)-linked primers to synthesize first-strand cDNA; (ii) addition of complementary primers to the template-switching oligo for template conversion; (iii) incorporation of fusion primers with barcodes to amplify cDNA products; (iv) purification of cDNA using ProNex Beads and quantification with Qubit fluorometer (Invitrogen) (v) SMRTbell library construction using the 2.0 kit; and (vi) removal of single-stranded overhangs, DNA damage repair, end repair, A-tailing, and adapter ligation. Libraries were purified with 0.45× AMPure PB beads, size-selected for 1–10 kb fragments, evenly loaded onto SMRT Cells, and sequenced on the PacBio Sequel II/IIe system (Pacific Biosciences, CA, USA).

### Genome size estimation

The genome size and heterozygosity of the target plants were estimated using k-mer analysis. Illumina sequencing data quality was assessed with FastQC (v0.11.9, https://github.com/s-andrews/FastQC). The frequency distribution of 17-mer segments from the short reads was calculated using Jellyfish (v2.3.0) [46], and genome size and heterozygosity were subsequently estimated with GenomeScope (v1.0.0) [47].

To experimentally validate the genome size, flow cytometry analysis was performed using fresh young leaves of CSLZ and *O. sativa* ssp. *japonica* (~380 Mb) as the internal reference standard. Nuclei were extracted and stained using the CyStain UV Precise P kit (Sysmex Partec, Germany) following the manufacturer’s instructions. Briefly, leaf tissues of the sample and the standard were co-chopped in Nuclei Extraction Buffer to release nuclei, filtered through a nylon mesh, and stained with the Staining Buffer containing 4′,6-diamidino-2-phenylindole. The relative fluorescence intensity of the nuclei was measured using a Partec CyFlow Space flow cytometer (Sysmex Partec, Germany) equipped with a UV LED/lamp. The genome size of CSLZ was calculated based on the ratio of the G1 peak mean fluorescence intensity of the sample to that of the internal standard.

### *De novo* assembly and quality assessment of haplotype genomes

A hybrid assembly pipeline was implemented to construct the phased diploid genome (Fig. 1B). To maximize the structural utility of deep long-read coverage, PacBio CLR reads (~94.72×) were first assembled using FALCON (v0.3.0) [48], polished with Arrow and Filon [49], and scaffolded with MboI Hi-C data using the Juicebox and 3D-DNA pipeline [50] to generate the primary haploid reference (Haploid Genome V1). Simultaneously, PacBio CCS (~39×) and HindIII Hi-C data were assembled using hifiasm (v0.16.1-r375). Specifically, hifiasm integrates Hi-C linkage information to partition the assembly graph into haplotype-resolved string graphs by distinguishing *cis*-chromosomal contacts from *trans*-interactions. These phased contigs were subsequently anchored to the CLR-based primary reference using RagTag (v2.0.1) [51]. Finally, Hi-C interaction matrices were generated using HiC-Pro (v3.1.0) [52], and the assembly underwent manual curation in Juicebox to correct misassemblies and finalize the order and orientation of the phased chromosomes (Phased Genome V2).

Genome assembly quality and completeness were evaluated using Merqury (v1.3) [53], BUSCO (v5.5.0) [54], and Kraken2 (v2.17.1) [55]. For base-level accuracy and phasing consistency, a k-mer database (*k* = 21) was constructed from PacBio HiFi reads using Meryl (v1.3), and consensus QV scores and k-mer completeness were calculated using Merqury. Gene space completeness was assessed using BUSCO with the eudicots_odb10 lineage dataset. To detect potential contamination, taxonomic classification was performed on both the assembled chromosomes and unmapped reads using Kraken2 against the Standard-8 database (encompassing RefSeq archaea, bacteria, plasmid, viral, human, and UniVec_Core sequences). A strict confidence threshold of 0.005 (--confidence 0.005) was applied to assembly classification.

### Repetitive sequence and gene annotation

Gene model prediction was performed by integrating three approaches: transcript-based prediction, homologous protein-based prediction, and *de novo* prediction. Both haplotype genomes were processed separately using GETA (https://github.com/chenlianfu/geta). The workflow included the following steps:

(1) The genome was analyzed with itself using RepeatModeler (v1.0.11) [56] to identify high-frequency repeat sequences and construct a species-specific repeat database. For the detailed analysis of TEs and genomic repetitiveness, we employed the EDTA pipeline (v1.9.6), which integrates multiple *de novo* tools (including LTR_FINDER, LTR_harvest, and TIR-Learner) to produce a high-quality, non-redundant TE library. To capture specific repeat classes with higher sensitivity, we additionally employed MITE-Hunter [57] to identify miniature inverted-repeat TEs (MITEs) and TRF [58] to annotate tandem repeats (parameters: 2 7 7 80 10 50 500 -f -d -m). The *de novo* libraries from RepeatModeler, MITE-Hunter, and TRF were merged to create a unified database. The genome was then aligned to this comprehensive repeat database using RepeatMasker (v1.331) [56] to identify repetitive elements. The combined results ensured a robust annotation of genomic repeats, which were subsequently masked to facilitate accurate gene prediction.

(2) Raw transcriptome data were filtered with Trimmomatic (v0.33) [59] to remove low-quality reads and adapter sequences. Filtered reads were aligned to the genome using HISAT2 (v2.2.1) [60]. Coverage thresholds were applied based on sequencing depth to obtain reliable transcripts and intron information. High-confidence transcripts were analyzed with TransDecoder (v5.50, http://github.com/TransDecoder/TransDecoder) to generate a transcriptome-based gene model.

(3) A high-quality homologous protein database was constructed using annotated sequences from *P. andersonii, T. orientalis,* hops (*H. lupulus*), *A. thaliana*, and CSLZ haploid protein sequences. Gene models from the transcript-based step were aligned to this database using GeneWise (https://www.ebi.ac.uk/birney/wise2/) to identify homologous genes.

(4) AUGUSTUS was used to perform *de novo* gene prediction, informed by intron, exon, start codon, and stop codon information obtained from transcript-based and homology-based predictions. Finally, results from all three methods were integrated to produce accurate, high-quality gene models for each haplotype genome.

Genome complexity analysis to visualize the landscape of sequence repetitiveness and distinguish between local and long-range complexity, we employed the alignment-free method AlcoR [61]. Complexity maps were generated for each haplotype using two configurations: a ‘local’ model (order 5, window 10) to detect short-range repetitiveness, and a ‘distant’ model (order 12, window 50) to capture broader repetitive structures such as TEs and segmental duplications. The resulting complexity scores were plotted along the chromosomes to identify regions of high and low complexity.

### Gene family and genome evolution analysis

Haploid genome annotations from nine reference species, *O. sativa, S. lycopersicum, V. vinifera, A. thaliana, M. notabilis, F. macrocarpa, P. andersonii, T. orientale*, and *H. lupulus* were collected, together with the *Cannabis sativa* (CSLZ) haploid genome annotation generated in this study. In total, 10 species were included for comparative genomic analyses. The annotated datasets for *O. sativa, S. lycopersicum,* and *A. thaliana* were obtained from the Phytozome database (V7.0, ITAG 4.0, and TAIR10, respectively). The annotation of *V. vinifera* was retrieved from EnsemblPlants (PN40024.v4). Genome annotations of *T. orientale, P. andersonii,* and *M. notabilis* were downloaded from NCBI (GCA_002914845.1, GCA_002914805.1, and GCF_000414095.1, respectively). The *H. lupulus* annotation was obtained from HopBase [62], and the *F. macrocarpa* annotation was derived from a previously published study (2020) [63].

For each species, the longest transcript per gene was extracted using in-house Python scripts. Orthogroups were identified using OrthoFinder v2.5.4 [64], and 606 single-copy orthologs were extracted to construct a phylogenetic tree using the maximum likelihood method implemented in IQ-TREE v2.2.0.3 [65]. The best-fit substitution model (Q.plant + F + R5) was selected automatically, and branch supports were evaluated with 1500 ultrafast bootstrap replicates (−bb 1500). Four-fold degenerate transversion sites were extracted using ParaAT v2.0 [66], and species divergence times were estimated with the MCMCtree program in PAML v4.9j [67], calibrated using divergence time data from the TimeTree database [68]. Gene family expansion and contraction were analyzed with CAFE5 [69], and phylogenetic trees were visualized using EvolView [70]. KEGG enrichment of expanded gene families was conducted using ClusterProfiler [71].

Speciation and WGD events were inferred based on the distribution of synonymous substitution rates (Ks) among orthologous gene pairs (*C. sativa* vs. *H. lupulus*, *F. macrocarpa*, and *V. vinifera*) and paralogous pairs within the *C. sativa* genome. Syntenic blocks were identified using MCScan (Python version) [72], and gene pairs within these blocks were classified as orthologs or paralogs. Ks values were calculated using the WGD pipeline v1.1 [73].

### Structural variant identification

Pairwise genome alignments were performed between the *C. sativa* CSLZ_hapB assemblies and the CSLZ_hapA reference using Minimap2 [74]. SVs were identified from the pairwise alignments using paftools, a utility included in the Minimap2 package. The alignment results were visualized as genome-wide dot plots using dotPlotly (https://github.com/tpoorten/dotPlotly/) and Plotsr (https://github.com/schneebergerlab/plotsr). Detected SVs were categorized based on their type and size, and only high-confidence variants were retained for downstream analysis.

### Identification of allelic variations and allele-specific expressed genes.

Alleles were defined using a combination of synteny-based and coordinate-based approaches. Syntenic blocks between the two haplotypes were identified using MCScanX [72], and gene pairs with high sequence similarity within each block were designated as allele A and allele B. Gene models sharing identical coding sequences were merged and considered a single allele. For genes not located within syntenic blocks, sequence alignment against the monoploid assembly was performed using GMAP [75], and two genes were considered potential alleles if their genomic coordinates overlapped by more than 50%.

To minimize reference mapping bias, RNA-seq reads were quality-trimmed using Trimmomatic [59], and mapped to the combined diploid reference assembly (containing both Haplotype A and Haplotype B sequences) using Bowtie [76]. This competitive mapping strategy ensures that reads originating from heterozygous sites are preferentially aligned to their true haplotype origin based on sequence divergence (SNPs and Indels) and optimal alignment scores, thereby minimizing cross-mapping between homoeologs. Transcript abundance was quantified using RSEM [77], which further ensures quantification reliability by handling read mapping uncertainty through an expectation–maximization algorithm. Differential expression analysis was performed using edgeR v4.0.16 [78]. Genes with low expression (read counts <10 in all samples) were filtered out prior to analysis. For ASE analysis, only sites with a minimum coverage of 20 reads and at least 5 reads supporting each allele were retained to ensure statistical power. ASE was identified using a Binomial test followed by FDR correction. Genes were considered ASEGs if they met the following criteria: (i) Adjusted *P*-value (FDR) < 0.05 and (ii) log_2_|Fold Change| (ratio of Allele A/Allele B expression) > 1.

Functional enrichment analyses of ASE genes were conducted using clusterProfiler [71] for GO and KEGG pathways. Heatmaps of gene expression were generated using pheatmap (https://github.com/raivokolde/pheatmap), and PCA was performed using PCAtools (https://github.com/kevinblighe/PCAtools). Venn diagrams of gene set overlaps were generated with E Venn [79]. Visualization of TE insertions between haplotypes was carried out using IGV [80], and *cis*-regulatory elements in promoter regions were identified based on the PlantCARE database [81].

### Transient expression in *N. benthamiana* and metabolite detection

For transient expression, recombinant pEAQ (a virus-based plant expression vector) plasmids were introduced into *Agrobacterium tumefaciens* strain GV3101 via transformation. Positive colonies verified by colony PCR were cultured in Luria-Bertani (LB) medium containing appropriate antibiotics until the OD_600_ reached 0.5–0.7. The bacterial cells were harvested and resuspended in infiltration buffer to an OD_600_ of 0.6–0.7. Strains carrying different gene constructs were mixed in equal proportions and incubated with 200 μM acetosyringone in the dark for 2–3 h before infiltration into leaves of 3–4-week-old *N. benthamiana* plants. Following infiltration, plants were incubated in the dark for 24 h and then transferred to a greenhouse under normal growth conditions for an additional 3 days. Substrate solutions were infiltrated on the fourth day, and leaf tissues were harvested after another 24 h of dark incubation, lyophilized, and stored for subsequent metabolite analysis.

For metabolite detection, ~40 mg of lyophilized leaf powder was saponified and extracted with 2 ml of 50% ethanol containing 20% KOH at 100°C for 10 min, with 5α-cholestane used as an internal standard. Metabolites were extracted twice with *n*-hexane, and the combined organic phases were concentrated and derivatized with pyridine and N,O-bis(trimethylsilyl)trifluoroacetamide (BSTFA) containing 1% trimethylchlorosilane (TMCS) at 70°C for 1 h. The derivatized samples were then evaporated to dryness, redissolved in chloroform, and filtered prior to Gas chromatography-mass spectrometry (GC–MS) analysis. GC-MS was using an Agilent 8890 Gas Chromatograph coupled to an Agilent 7000E Triple Quadrupole Mass Spectrometer. Metabolites were identified by comparison with the NIST14 mass spectral library and relatively quantified by normalizing peak areas to the internal standard.

## Supplementary Material

Web_Material_uhag159

## Data Availability

The data that support the findings of this study have been deposited in both the China National Center for Bioinformation (CNCB) and the National Center for Biotechnology Information (NCBI). Raw sequencing data: The raw resequencing data generated in this study have been deposited in the CNCB Genome Sequence Archive (GSA) under the accession number CRA033206, and in the NCBI BioProject database under the accession number PRJNA1417719. Genome assembly data: The haplotype-resolved CSLZ genome assemblies have been deposited in the CNCB Genome Warehouse (GWH) under the accession numbers GWHGZJI00000000.1 (HapA) and GWHGZJJ00000000.1 (HapB). Additionally, the assemblies have been submitted to NCBI GenBank under the accession numbers JBVFIV000000000 (HapA) and JBVFIW000000000 (HapB). Custom Code: The custom scripts and pipelines used in this study are publicly available on GitHub at https://github.com/caiisen/LZ_phased_genome.
