# Supplementary material for: Haplotype-resolved regulation of secondary metabolism in cannabis
Source: Hortic Res. 2026 Apr 28;13(9):uhag159. doi: 10.1093/hr/uhag159 (PMC13522725; doi:10.1093/hr/uhag159)
Supplement: Supplementary file 1 [file web_material_uhag159.zip › Supplementary_Figures.pdf]

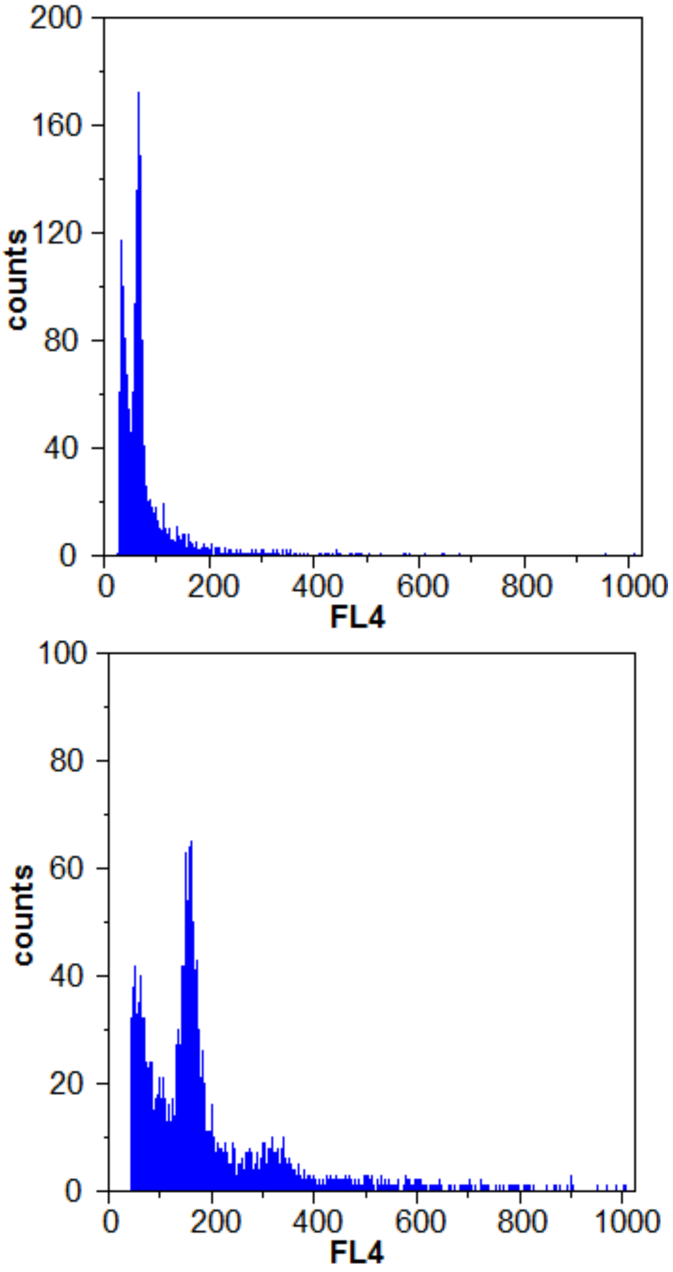

FigS1. Genome size estimation using flow cytometry. Histogram of fluorescence intensity for nuclei isolated from the internal reference *Oryza sativa* ssp. japonica (up) and CSLZ (down).

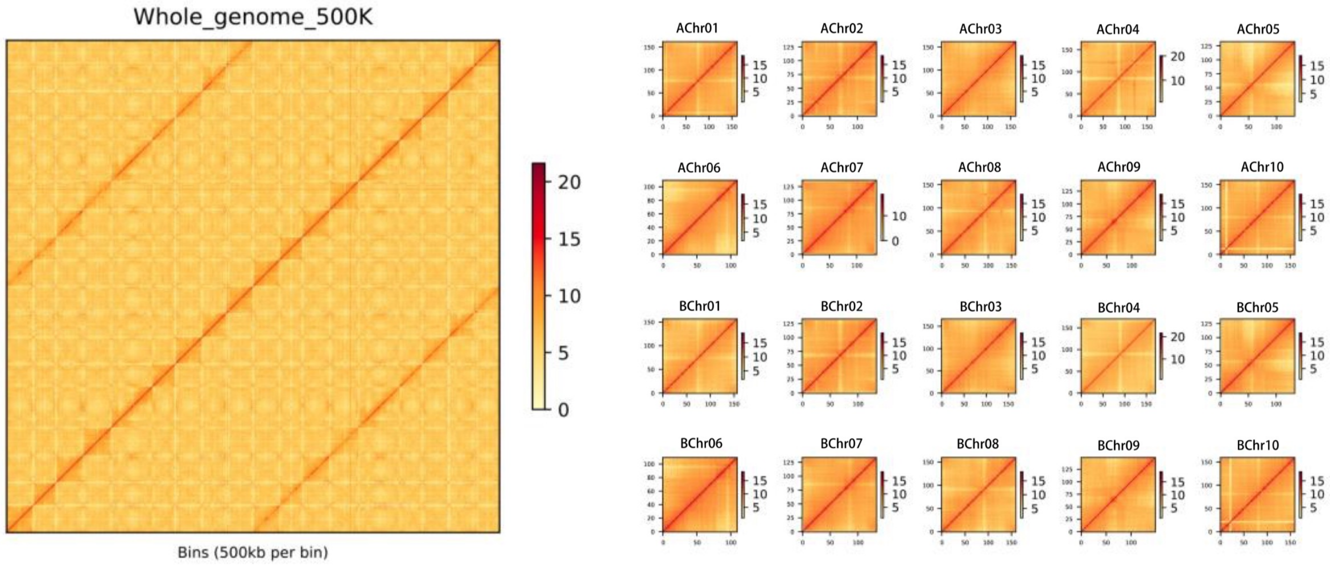

FigS2. Heatmap of the intensity signal of Hi-C chromosome interactions (bin=200 kb)

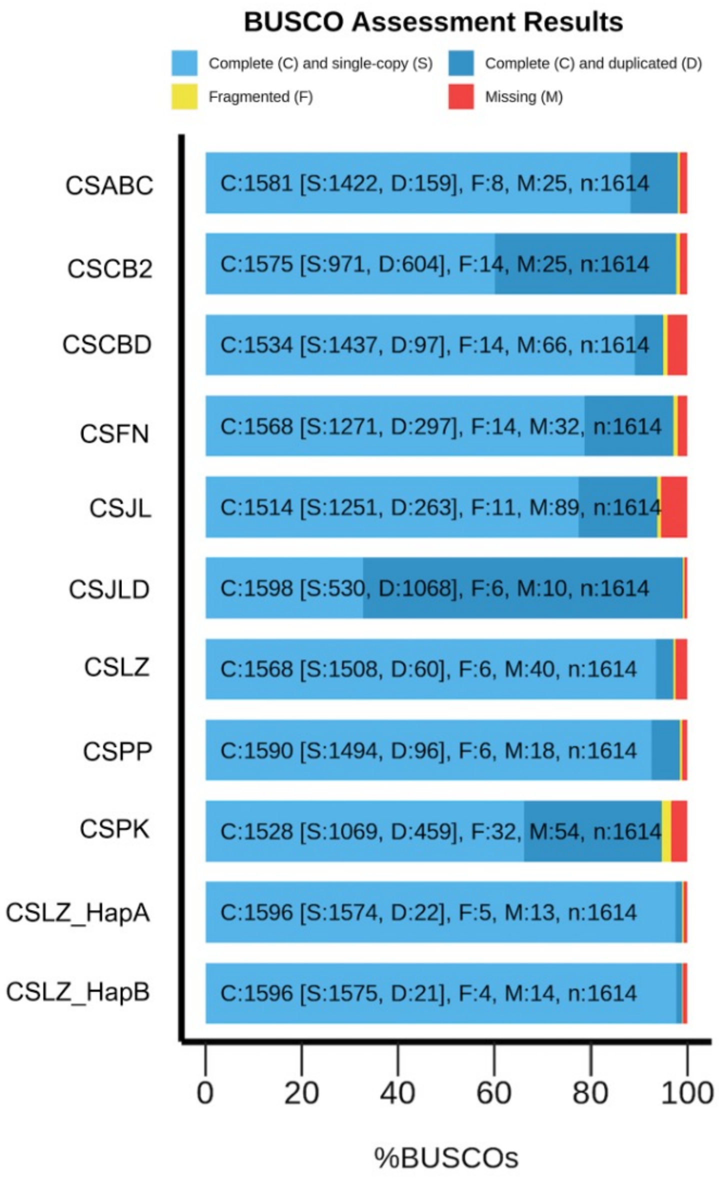

FigS3. BUSCO assessment results for CSLZ and other cannabis genomes

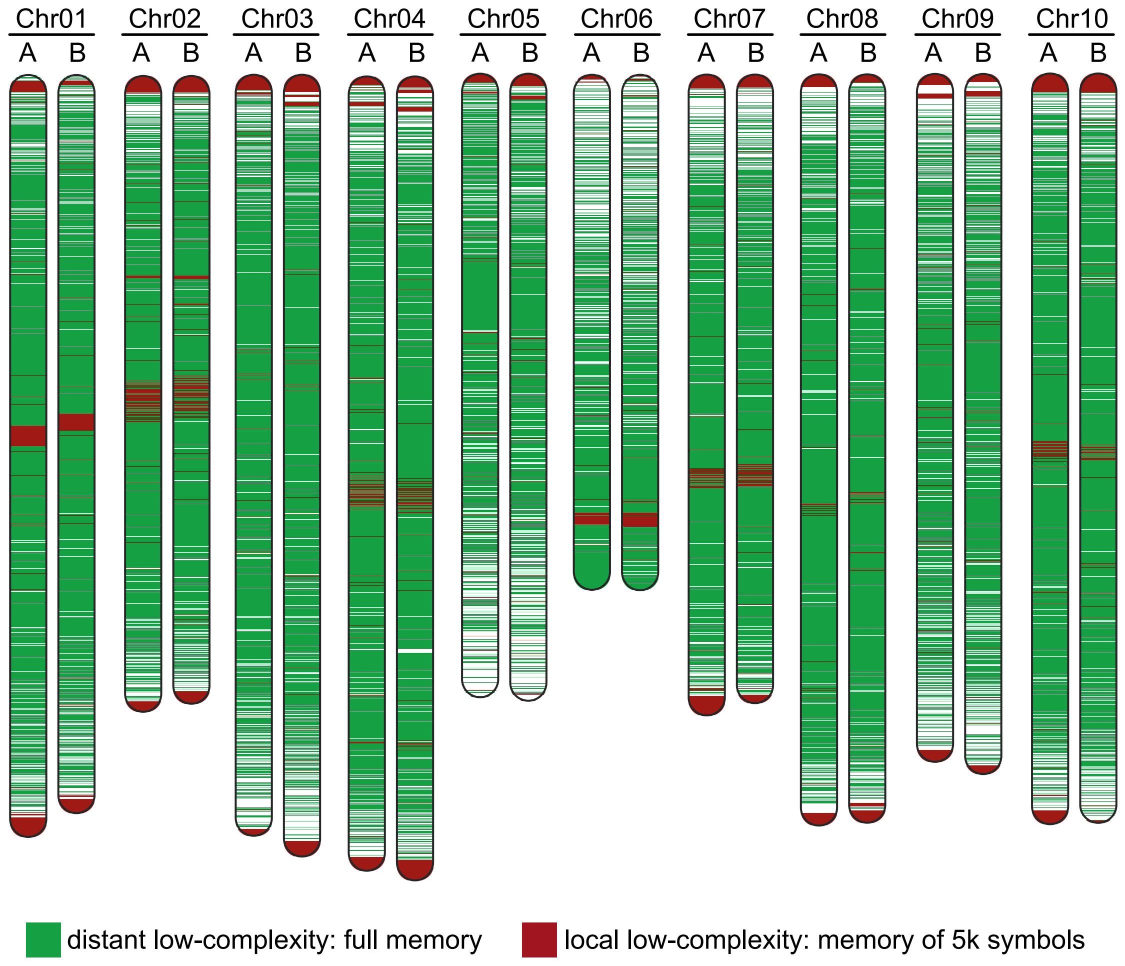

FigS4. Chromosome low-complexity (redundancy) map using different memories. The green color stands for the identification of LCRs using a setup of a model that uses the full memory, while the red color uses a memory of p = 5,000 symbols. In the map, the regions with the red color overlap the green; therefore, regions marked with red are also green, and the green regions that are visible stand for low complexity related to distant patterns.

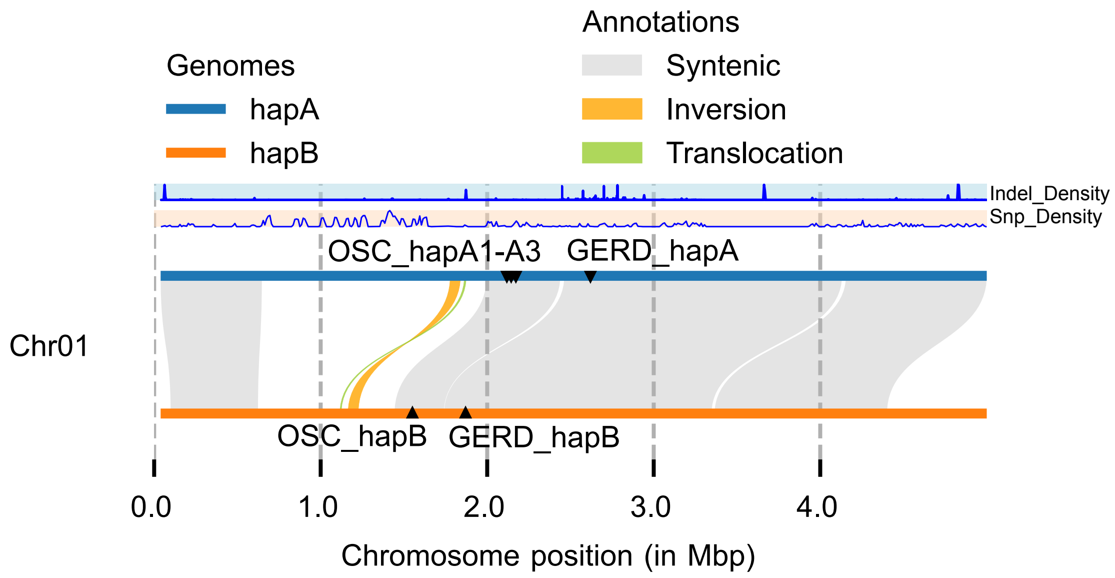

FigS5. Linear visualization of structural variants and gene synteny at the *GERD* and *OSC* loci.

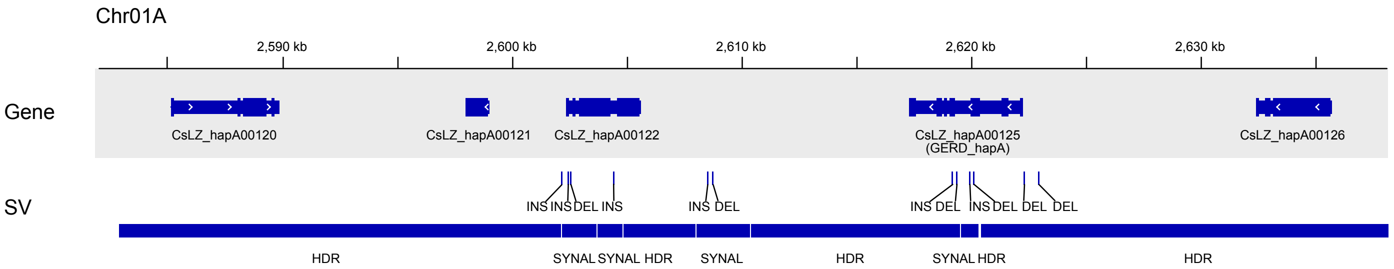

FigS6. Linear visualization of structural variants and gene synteny at the *GERD_hapA* loci. INS: insertion in query, DEL: deletion in query, HDR: highly diverged regions, SYNAL: alignment in syntenic region.

```
CsLZ_hapA0012      1 MSPCEDTIDEKRPNMPKFTPTIWGDYFMSSASNHHHSSLMETMENNNKES     50
                                   |||||||||||||||.||: ||||||||||||||||
CsLZ_hapB0013      1 --------------MPKFTPTIWGDYFMSHASS-HHSSLMETMENNNKES     35

CsLZ_hapA0012     51 YEKIIEMKEQVKNKLLHGLHPLENPLETLEYIDDIQRLGLSYYFENEIEQ    100
                     ||||||||||||||||||||||||||||||||||||||||||||||||||
CsLZ_hapB0013     36 YEKIIEMKEQVKNKLLHGLHPLENPLETLEYIDDIQRLGLSYYFENEIEQ     85

CsLZ_hapA0012    101 IL-EQFHNNYQNLIDFGDNNLYADALCFRLLRQQGYNIACDIFDKYKNEK    149
                     :| :||||| ||||||||||||||||||||||||||||||||||||||||
CsLZ_hapB0013     86 VLMKQFHNN-QNLIDFGDNNLYADALCFRLLRQQGYNIACDIFDKYKNEK    134

CsLZ_hapA0012    150 GKFKESISSDIRGMLNLYEAAQMRVHGEKILDEALIFTTTHLESSVKTCQ    199
                     |||||||.||||||||||||||||||||||||||||||||||||||||||
CsLZ_hapB0013    135 GKFKESILSDIRGMLNLYEAAQMRVHGEKILDEALIFTTTHLESSVKTCQ    184

CsLZ_hapA0012    200 LSSPYLDLVKHALMHPIRKSLQRREARLYISLYHQLPSHEEILLTLAKLD    249
                     ||||||||||||||||||||||||||||||||||||||||||||||||||
CsLZ_hapB0013    185 LSSPYLDLVKHALMHPIRKSLQRREARLYISLYHQLPSHEEILLTLAKLD    234

CsLZ_hapA0012    250 FNLLQKLHQKELSYITRWWKEFDYKSKHSFIKDRIVECYFWVYGVFFEAE    299
                     ||||||||||||||||||||||||||||||||||||||||||||||||||
CsLZ_hapB0013    235 FNLLQKLHQKELSYITRWWKEFDYKSKHSFIKDRIVECYFWVYGVFFEAE    284

CsLZ_hapA0012    300 TSQIRLIITKLIAILTIIDDAYDSFGTLEELEPFTQAIERWDICAIDTLP    349
                     ||||||||||||||||||||||||||||||||||||||||||||||||||
CsLZ_hapB0013    285 TSQIRLIITKLIAILTIIDDAYDSFGTLEELEPFTQAIERWDICAIDTLP    334

CsLZ_hapA0012    350 EYMKIFYMKLLEIYNEIEQFSKEKSYCPSYAKKGVQSLIRAYFKEAKWLH    399
                     |||||||||||||||||||||||:||||||||||||||||||||||||||
CsLZ_hapB0013    335 EYMKIFYMKLLEIYNEIEQFSKERSYCPSYAKKGVQSLIRAYFKEAKWLH    384

CsLZ_hapA0012    400 TKYIPTLDEYMPVGVDSAGSFMLISMVFIGMGDIVTKHSMDWIFSNPQPK    449
                     ||||||||||||||||||||||||||||||||||||||||||||||||||
CsLZ_hapB0013    385 TKYIPTLDEYMPVGVDSAGSFMLISMVFIGMGDIVTKHSMDWIFSNPQPK    434

CsLZ_hapA0012    450 IIQTMAIVGRVMNDIGYHKSERKKSSGEIVASTVECYMKQYGVPGEEAIE    499
                     |||||||||||||||||||||||||||||||||||||||||||.||||||
CsLZ_hapB0013    435 IIQTMAIVGRVMNDIGYHKSERKKSSGEIVASTVECYMKQYGVTGEEAIE    484

CsLZ_hapA0012    500 KLSEQVKDSWKDLNEDLLNPITIPRPLLMQVLKLVRVNHEIYREGDGFTQ    549
                     |||:||||||||||||||||||||||||||||||||||||||||||||||
CsLZ_hapB0013    485 KLSQQVKDSWKDLNEDLLNPITIPRPLLMQVLKLVRVNHEIYREGDGFTQ    534

CsLZ_hapA0012    550 PTLLKNLIHSLIINPIDF*    568
                     ||||||||||||||||||
CsLZ_hapB0013    535 PTLLKNLIHSLIINPIDF*    553
```

FigS7. Pairwise sequence alignment between GERD_HapA and GERD_HapB.

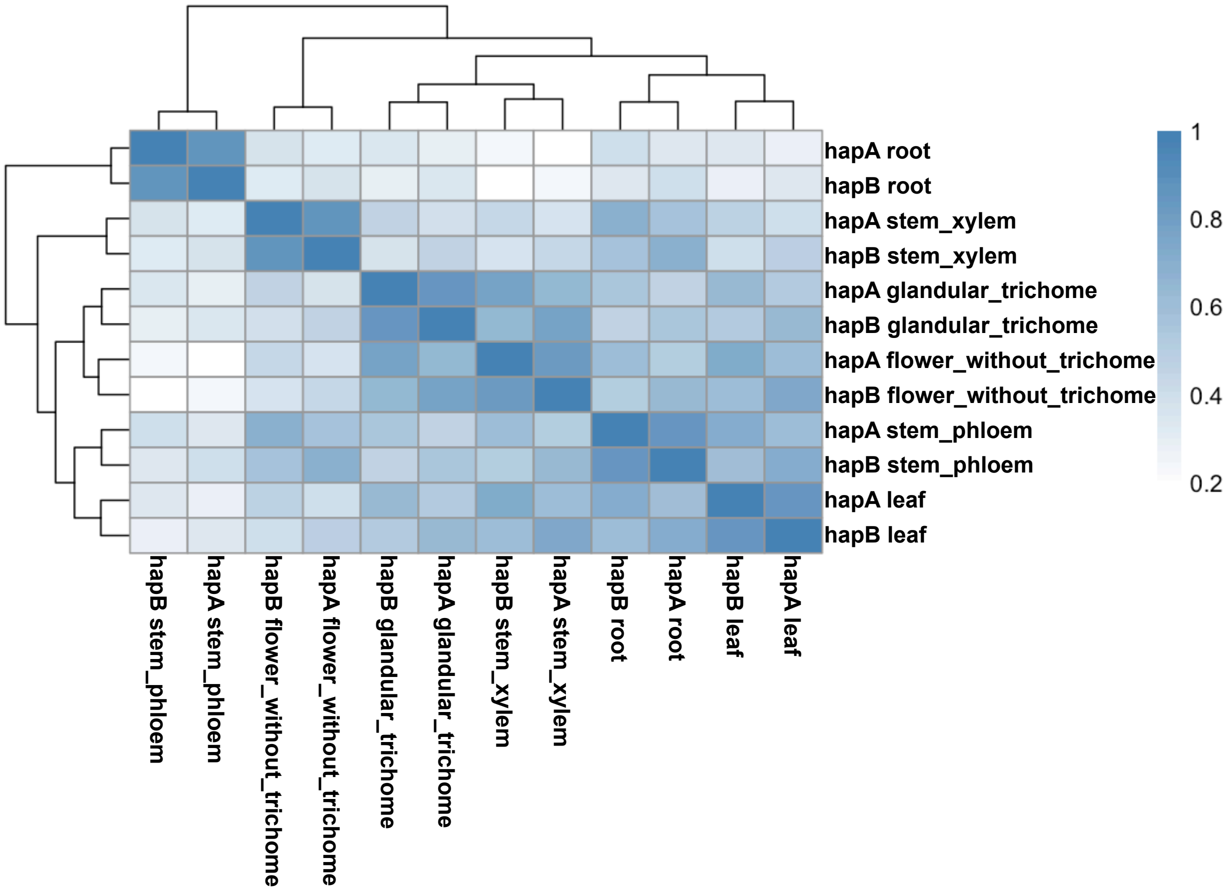

FigS8. Correlation analysis between roots, stem xylem, stem phloem, leaves, female flowers (without trichomes), and glandular trichomes.

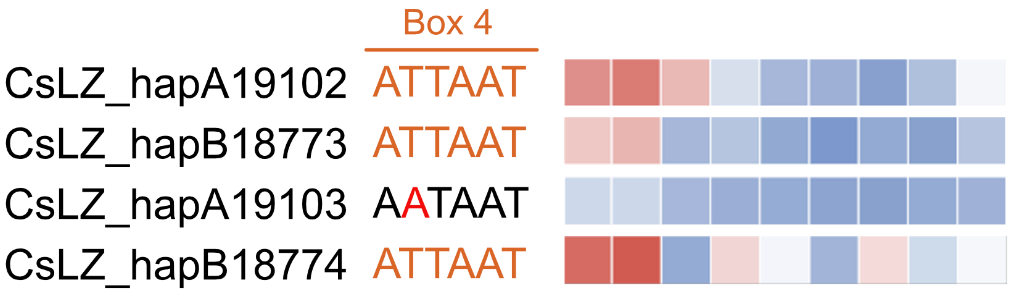

FigS9. Differences in HCT expression and promoter region in different haplotypes.

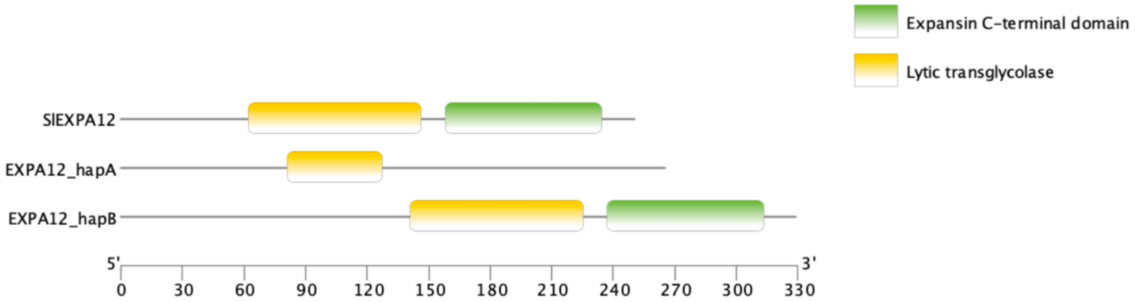

FigS10. Domains of SlEXPA12, EXPA12_hapA and EXPA12_hapB.
